# NIR-enzymatic dual-engine nanomotors overcome cartilage matrix barriers and reprogram the osteoarthritic microenvironment

**DOI:** 10.1126/sciadv.aej0289

**Published:** 2026-11-13

**Authors:** Cong Xu, Yuxin Zhang, Yalin He, Haiying Liang, Hong Wang, Yongxi Long, Jingyue Pan, Yicheng Ye, Yunshi Li, Yuejun Jiang, Weilin Lu, Lihan Shen, Yingfeng Tu, Yingjia Li

**Affiliations:** ^1^ Department of Medicine Ultrasonics, Nanfang Hospital, Southern Medical University, Guangzhou 510515, China.; ^2^ State Key Laboratory of Multi-organ Injury Prevention and Treatment, Guangdong Provincial Key Laboratory of New Drug Screening & Guangdong-Hong Kong-Macao Joint Laboratory for New Drug Screening, School of Pharmaceutical Sciences, Southern Medical University, Guangzhou 510515, China.; ^3^ Department of Ultrasound, First Affiliated Hospital of Guangzhou Medical University, Guangzhou 510120, China.; ^4^ The First School of Clinical Medicine, Southern Medical University, Guangzhou 510515, China.; ^5^ The Second School of Clinical Medicine, Southern Medical University, Guangzhou 510280, China.; ^6^ Department of Critical Care Medicine, Dongguan Institute of Respiratory and Critical Care medicine, The Tenth Affiliated Hospital, Southern Medical University (Dongguan People’s Hospital), Dongguan 523059, China.

## Abstract

Restoring immune microenvironment homeostasis is critical for slowing osteoarthritis (OA) progression. However, the dense, avascular cartilage extracellular matrix restricts therapeutic access to deep inflammatory lesion sites. To address this challenge, we developed a self-propelled asymmetric yolk-shell nanomotor (mPDA-C@M), which overcomes this transport barrier through a dual drive strategy. By coupling cerium(IV) oxide–mediated catalytic propulsion with mesoporous polydopamine (mPDA)–enabled thermophoretic motion, the system achieves enhanced active transport, in which near-infrared irradiation reinforces directional movement and drives the nanomotor into deep cartilage. Beyond improved tissue access, mPDA-C@M modulates the osteoarthritic microenvironment by combining reactive oxygen species scavenging with mild photothermal stimulation. This synergistic effect up-regulates HSP70 expression and restores anabolic and catabolic balance through suppression of nuclear factor κB/mitogen-activated protein kinase inflammatory signaling. In rat and rabbit models, this dual strategy reduced cartilage degeneration, promoted matrix synthesis, and improved joint repair. These findings establish active-matrix traversal as a strategy for microenvironment-directed therapy in OA and other diffusion-limited inflammatory tissues.

## INTRODUCTION

Osteoarthritis (OA) is a prevalent, multifactorial degenerative disorder affecting more than 600 million people globally, imposing substantial socioeconomic burdens (*1*, *2*). Although traditionally viewed as a “wear-and-tear” disease, emerging evidence underscores that the core pathology of OA derives from the disruption of immune microenvironment homeostasis (*3*, *4*). This imbalance triggers a cascade of synovial inflammation, pathological subchondral bone remodeling, and progressive articular cartilage destruction, ultimately leading to chronic pain and joint dysfunction (*5*, *6*). While current interventions alleviate symptoms, disease-modifying therapies capable of halting OA progression remain elusive (*7*). Therefore, there is an urgent need for strategies that can fundamentally restore this lost immune homeostasis to promote genuine cartilage regeneration.

Under physiological conditions, chondrocytes are embedded within an extracellular matrix (ECM) dense in collagen and proteoglycans, which maintains metabolic balance (*8*, *9*). In OA, the pathological up-regulation of catabolic enzymes, such as collagen-degrading matrix metalloproteinases [especially matrix metalloproteinase-13 (MMP13)] and aggrecanases including ADAMTS-4 and ADAMTS-5, triggers ECM degradation and diminishes glycosaminoglycan (GAG) levels (*10*, *11*). However, a critical challenge persists despite this degradation: While the superficial cartilage layer is eroded, the deeper zones containing viable chondrocytes remain densely packed and difficult to penetrate (*12*). This residual dense ECM acts as a diffusion barrier that prevents intraarticular therapeutics from effectively penetrating into deep cartilage lesions (*13*, *14*), where proinflammatory cytokines and reactive oxygen species (ROS) accumulate, sustaining a vicious cycle of oxidative damage and chronic inflammation (*15*–*18*). Therefore, overcoming the cartilage ECM barrier to achieve deep tissue delivery is a prerequisite for effective OA treatment.

To address this challenge, micro/nanomotors, often termed nanorobots, have emerged as a revolutionary delivery system. Unlike traditional nanoparticles (NPs) that rely on inefficient Brownian motion, nanomotors convert endogenous or exogenous energy into mechanical force, enabling active propulsion across biological barriers (*19*–*23*). Notably, previous studies from our group have demonstrated that arthritic-actuated nanomotors alleviate synovial inflammation and cartilage degradation under inflammatory conditions (*24*, *25*). However, although endogenous chemical-driven motors show promise, their propulsion is often limited by the fluctuating concentrations of chemical fuels (e.g., H_2_O_2_) in the joint (*24*, *26*, *27*), resulting in insufficient force to breach the dense deep-zone ECM. This limitation necessitates the development of an intelligent, dual-driven system. In this context, near-infrared (NIR) light offers an attractive exogenous driving source because of its favorable tissue penetration and spatiotemporal controllability, which enable precise regulation of thermophoretic propulsion (*28*–*30*). NIR activation creates a synergistic strategy: Self-thermophoresis promotes deep cartilage penetration, while the generated mild heat simultaneously exerts the mild photothermal therapy (MPTT; 41° to 43°C) effect to modulate the inflammatory microenvironment (*6*, *31*). This mild photothermal stimulation can further induce HSP70-associated cytoprotection and suppress nuclear factor κB (NF-κB) signaling, thereby protecting chondrocytes from inflammatory injury and enhancing therapeutic efficacy (*32*–*34*).

In this work, we engineered a dual-energy–driven nanomotor with an asymmetric yolk-shell structure (mPDA-CeO_2_@Motor, abbreviated as mPDA-C@M) to achieve active cartilage ECM permeation for synergistic immune restoration and regeneration (Fig. 1). The system comprises a CeO_2_-anchored mesoporous polydopamine (mPDA) core driven by two mechanisms: (i) catalase (CAT)–like activity that decomposes endogenous H_2_O_2_ for chemical propulsion and (ii) NIR-triggered thermophoresis for directional movement. This dual-drive design empowers the nanomotors to actively “drill” into the dense collagen network, markedly enhancing deep tissue accumulation compared to passive counterparts. Upon reaching the deep zone, the internalized mPDA-C@M acts as an “immune rectifier.” It reprograms the oxidative and inflammatory microenvironment by scavenging ROS and polarizing macrophages from the proinflammatory M1 to the anti-inflammatory M2 phenotype. Furthermore, the synergistic action of MPTT and ROS scavenging up-regulates HSP70, promotes chondrocyte proliferation, and facilitates osteogenic differentiation. In both rat and rabbit OA models, mPDA-C@M demonstrated superior efficacy, validating the concept that “active penetration enables effective immune restoration.”

**Fig. 1.**
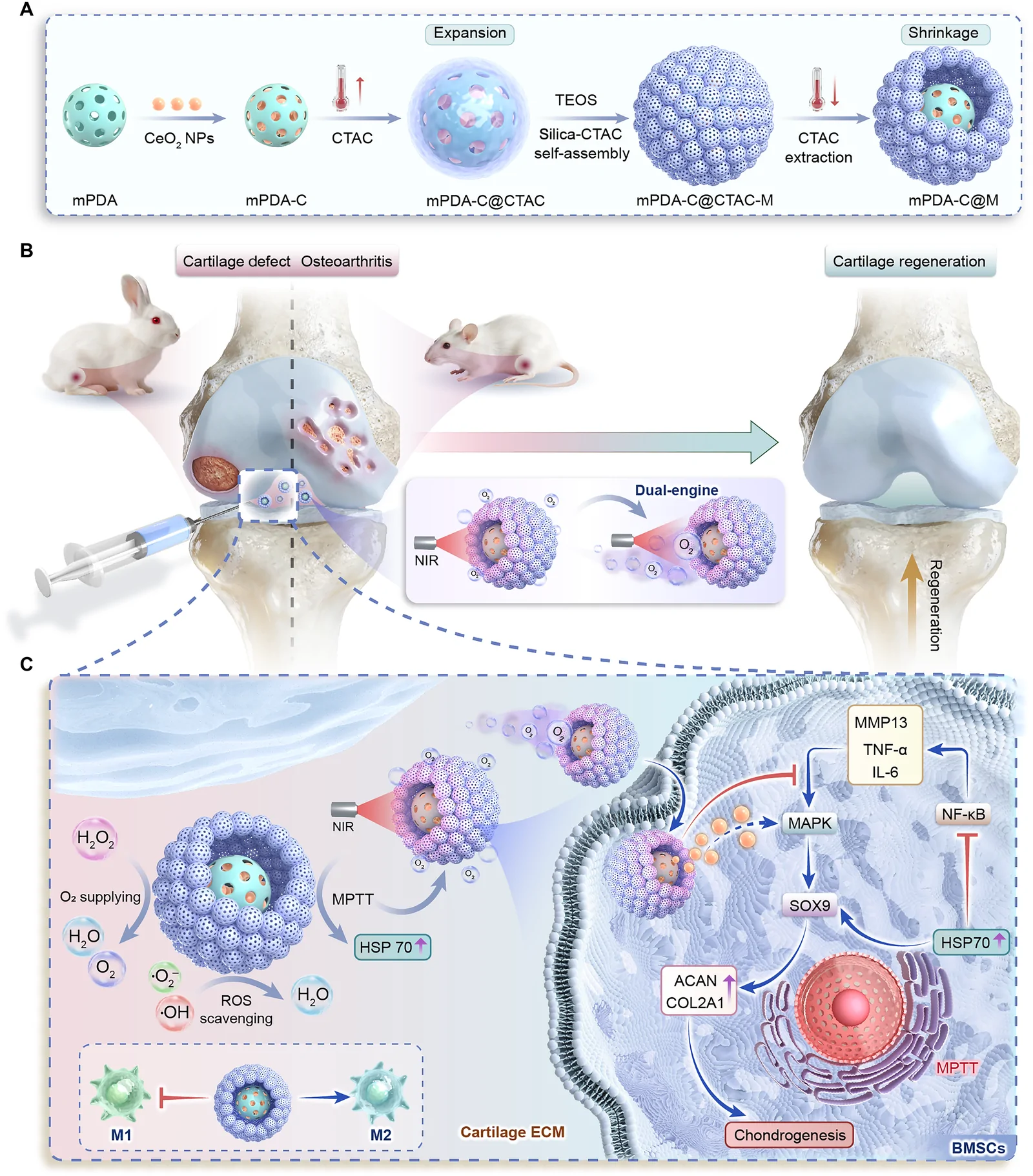
Schematic illustration of motion-potentiated nanomotors for active cartilage penetration and immune microenvironment reprogramming in OA. The mPDA-C@M is engineered with an asymmetric yolk-shell architecture designed to overcome the dense cartilage ECM barrier. It achieves inflammation-responsive self-propulsion via enzymatic oxygen generation and thermophoretic motion under NIR irradiation, synergistically penetrating the cartilage ECM barrier. Furthermore, upon NIR activation, the nanomotors up-regulate HSP70 via MPTT and scavenge ROS. This dual action regulates the metabolic balance, inhibits inflammation, and promotes the differentiation of bone marrow mesenchymal stem cells (BMSCs) for accelerating cartilage regeneration in the OA joint.

## RESULTS

### Preparation and characterization of mPDA-C@M

As illustrated in Fig. 2A, the fabrication of the nanomotors proceeded in two stages: the synthesis of CeO_2_-loaded mPDA (mPDA-C) and the subsequent construction of asymmetric yolk@shell nanomotors (mPDA-C@M) featuring a silica shell via an “expansion-shrinkage” strategy (*35*). Initially, mPDA spheres were synthesized using a formate and ethanol–water–1,3,5-trimethylbenzene (TMB)–Pluronic F-127 quaternary emulsion system. Separately, CeO_2_ NPs with a hydrodynamic diameter of 5.2 ± 0.5 nm were prepared via a precipitation method (fig. S1) (*36*). Notably, surface modification with DSPE–polyethylene glycol, molecular weight 2000 (PEG-2000) endowed the CeO_2_ NPs with excellent aqueous dispersibility, maintaining colloidal stability for at least 7 days. X-ray diffraction analysis confirmed the crystal structure of the synthesized CeO_2_ NPs. Subsequently, these CeO_2_ NPs were integrated with dimethylamino-modified mPDA to yield mPDA-C. Compared with pristine mPDA, the loading process increased the hydrodynamic diameter from 235.5 ± 6.5 to 251.2 ± 6.7 nm and reversed the surface potential from positive to negative (Fig. 2, B and C), confirming the effective encapsulation of CeO_2_ NPs within the mesopores of mPDA.

**Fig. 2.**
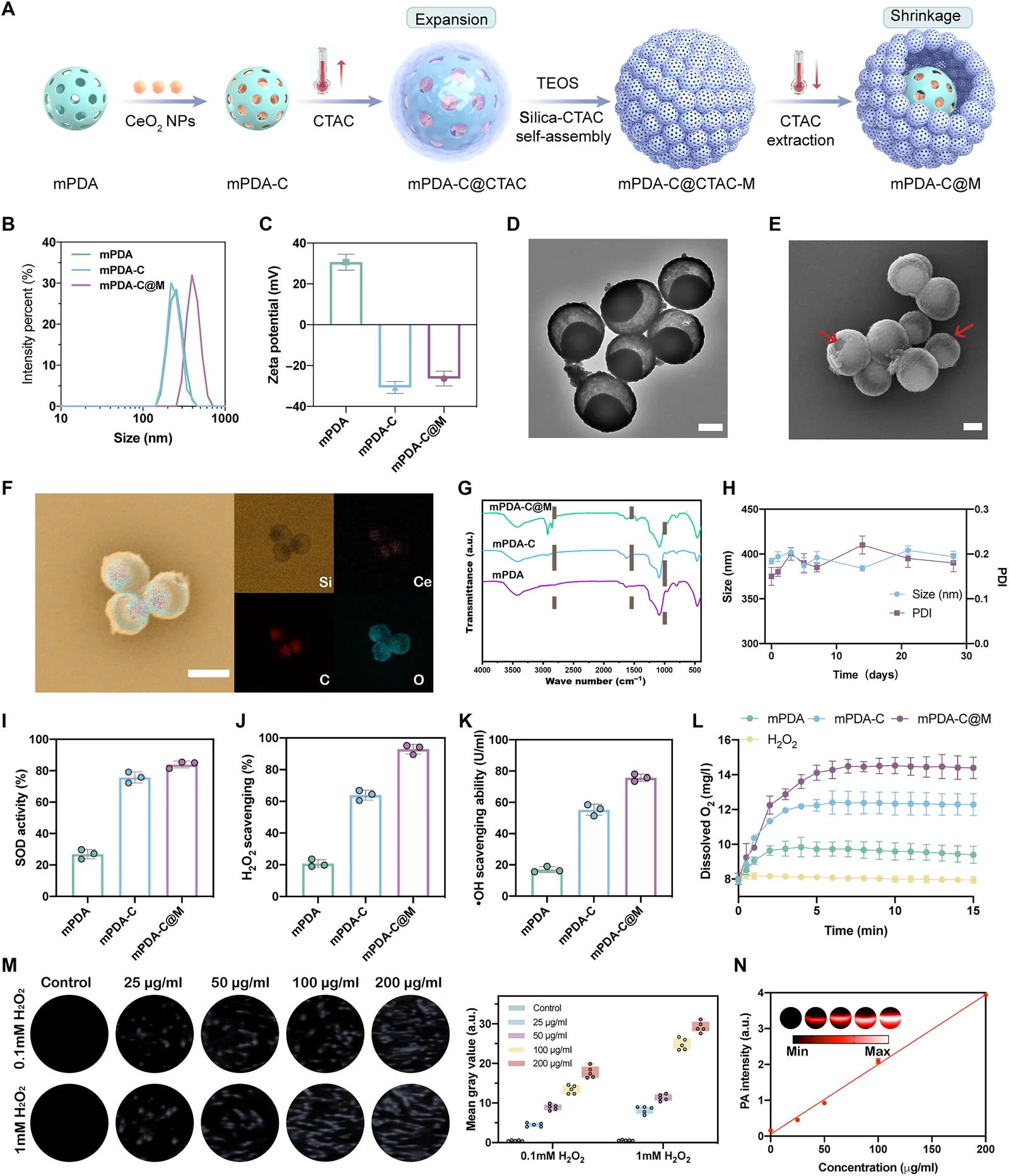
Synthesis and characterization of mPDA-C@M. (**A**) Schematic illustration of the synthesis process of mPDA-C@M. (**B**) Hydrodynamic size distribution of mPDA, mPDA-C, and mPDA-C@M. (**C**) Zeta potential of mPDA, mPDA-C, and mPDA-C@M (*n* = 3; mean ± SD). (**D**) Transmission electron microscopy (TEM) image of mPDA-C@M. Scale bar, 200 nm. (**E**) Scanning electron microscopy (SEM) image of mPDA-C@M. Scale bar, 200 nm. Red arrows indicate the lateral opening of the nanomotor. (**F**) SEM image of mPDA-C@M and the corresponding elemental mapping of Si, Ce, C, and O. Scale bar, 500 nm. (**G**) Fourier transform infrared (FTIR) spectra of mPDA, mPDA-C, and mPDA-C@M. a.u., arbitrary units. (**H**) Colloidal stability evaluation of mPDA-C@M in phosphate-buffered saline (PBS) (*n* = 3; mean ± SD). (**I**) Superoxide dismutase (SOD)–mimicking activity of mPDA, mPDA-C, and mPDA-C@M (*n* = 3; mean ± SD). (**J**) H_2_O_2_ scavenging and (**K**) ·OH scavenging capabilities of mPDA, mPDA-C, and mPDA-C@M (*n* = 3; mean ± SD). (**L**) O_2_ production efficiency of mPDA, mPDA-C, and mPDA-C@M (*n* = 3; mean ± SD). (**M**) In vitro ultrasound detection of oxygen generated by mPDA-C@M, with corresponding mean gray value intensity analysis (*n* = 5; mean ± SD). (**N**) Photoacoustic (PA) images of mPDA-C@M at increasing concentrations (excitation: 810 nm), with quantitative PA amplitude analysis (inset).

The yolk@shell mPDA-C@M with a lateral opening was then fabricated through solvent-mediated cetyltrimethylammonium chloride (CTAC) template extraction. During this process, the CTAC-adsorbed mPDA-C microspheres swelled at elevated temperatures, facilitating the coassembly of silica oligomers and CTAC into a mesoporous silica shell. Upon cooling, the mPDA-C core underwent thermal shrinkage, whereas the silica shell remained relatively rigid, thereby generating an internal void between the core and shell. Meanwhile, the anisotropic interfacial reconstruction during the expansion-shrinkage process led to the formation of a lateral opening, yielding the characteristic asymmetric yolk@shell structure. A notable size increase in mPDA-C@M, coupled with a shift in zeta potential from −30.7 to −26.4 mV, confirmed the successful coating of the silica shell (Fig. 2C). Morphological characterization via transmission electron microscopy (TEM) and scanning electron microscopy (SEM) (Fig. 2, D and E) revealed that mPDA-C@M has a uniform diameter of 396 ± 24 nm, a distinct asymmetric architecture, and a characteristic rough, porous silica shell. Energy-dispersive x-ray analysis (Fig. 2F) detected Si, Ce, C, and O on the surface, further verifying the silica layer formation. Fourier transform infrared (FTIR) spectroscopy (Fig. 2G) corroborated the composition: The peak at ∼1650 cm^−1^ (aromatic C═C/N─H stretching) indicated the presence of mPDA, while new peaks at 2850 to 2960 cm^−1^ (alkyl C─H stretching) and ∼1080 cm^−1^ (Si─O─Si asymmetric stretching) confirmed the silica coating. Furthermore, mPDA-C@M demonstrated exceptional long-term colloidal stability in phosphate-buffered saline (PBS), with no appreciable changes in hydrodynamic diameter or polydispersity index (PDI) over 30 days (Fig. 2H), satisfying a critical prerequisite for in vivo biological applications.

To evaluate the antioxidative efficacy of mPDA-C@M, we performed a series of in vitro assays targeting superoxide dismutase (SOD)–like and CAT-like activities, both of which are pivotal for scavenging ROS in the inflammatory microenvironment of OA (*5*, *37*). The SOD-mimicking activity was determined by the superoxide anion (O_2_^•−^) scavenging efficiency. As shown in Fig. 2I, mPDA-C@M achieved an O_2_^•−^ scavenging rate of 84%, markedly outperforming mPDA (29%) and mPDA-C (76%), thereby demonstrating its ability to catalyze the dismutation of O_2_^•−^ into H_2_O_2_ and O_2_. The CAT-mimicking activity was assessed by measuring H_2_O_2_ scavenging, hydroxyl radical (·OH) elimination, and O_2_ generation. H_2_O_2_, a persistent ROS in OA joints, was quantified using a Ti(SO_4_)_2_-based colorimetric assay. As presented in Fig. 2J, mPDA-C@M exhibited a H_2_O_2_ scavenging rate of 92.07%, 1.39-fold and 4.84-fold higher than that of mPDA-C and mPDA, respectively. Subsequent ·OH scavenging assays confirmed that mPDA-C@M has superior reactivity, likely enhanced by improved diffusion when H_2_O_2_ serves as a fuel (Fig. 2K). Consistent with these findings, O_2_ release from mPDA-C@M was concentration dependent (fig. S2), with more O_2_ generation compared to the control groups (Fig. 2L). Collectively, these results indicate that the unique asymmetric yolk@shell structure of mPDA-C@M not only preserves the intrinsic catalytic activity of the CeO_2_ nanozyme but also facilitates efficient reactant accessibility and mass transport, thereby enhancing its antioxidative performance in an inflammatory microenvironment.

On the basis of the robust oxygen-generating capability of mPDA-C@M, we further evaluated its potential as an echogenic agent using water-sac phantom tests. H_2_O_2_ solutions (0.1 and 1 mM) were used to simulate the pathological environment of arthritic joints, with 1 mM representing the approximate maximum H_2_O_2_ concentration reported under inflammatory conditions (*24*, *38*). As depicted in Fig. 2M, a distinct O_2_-derived echogenic signal was detectable even at 0.1 mM H_2_O_2_. The signal intensity increased with both H_2_O_2_ concentration and nanomotor dosage, attributable to enhanced O_2_ bubble production. These findings indicate that mPDA-C@M can convert pathological H_2_O_2_ into sufficient O_2_ bubbles for ultrasound visualization of inflammatory microenvironments. Moreover, mPDA-C@M exhibited a linear concentration-dependent photoacoustic (PA) signal in Fig. 2N, supporting its potential for quantitative PA imaging and treatment monitoring.

### Photothermal performance of mPDA-C@M

Leveraging the intrinsic photothermal conversion capability of the mPDA core, mPDA-C@M functions as an efficient heat generator under 808-nm NIR irradiation (*39*). Finite element simulations confirmed that under 808-nm NIR irradiation, the asymmetric yolk@shell structure of mPDA-C@M generates a nonuniform temperature distribution across the nanomotor surface, leading to a pronounced local thermal gradient that is essential for self-thermophoretic propulsion (Fig. 3A). Experimentally, aqueous dispersions of mPDA-C@M exhibited a rapid, concentration-dependent thermal response (Fig. 3B). For instance, irradiation of a suspension (150 μg/ml; 1.0 W/cm^2^ for 10 min) triggered a substantial temperature increase (Δ*T*) of ≈22.1°C, vastly exceeding the negligible rise (2.1°C) observed in the PBS control. The heating rate and maximum temperature were positively correlated with both nanomotor concentration (0 to 150 μg/ml) and laser power density (0.2 to 1.5 W/cm^2^) (Fig. 3, C and D, and fig. S3).

**Fig. 3.**
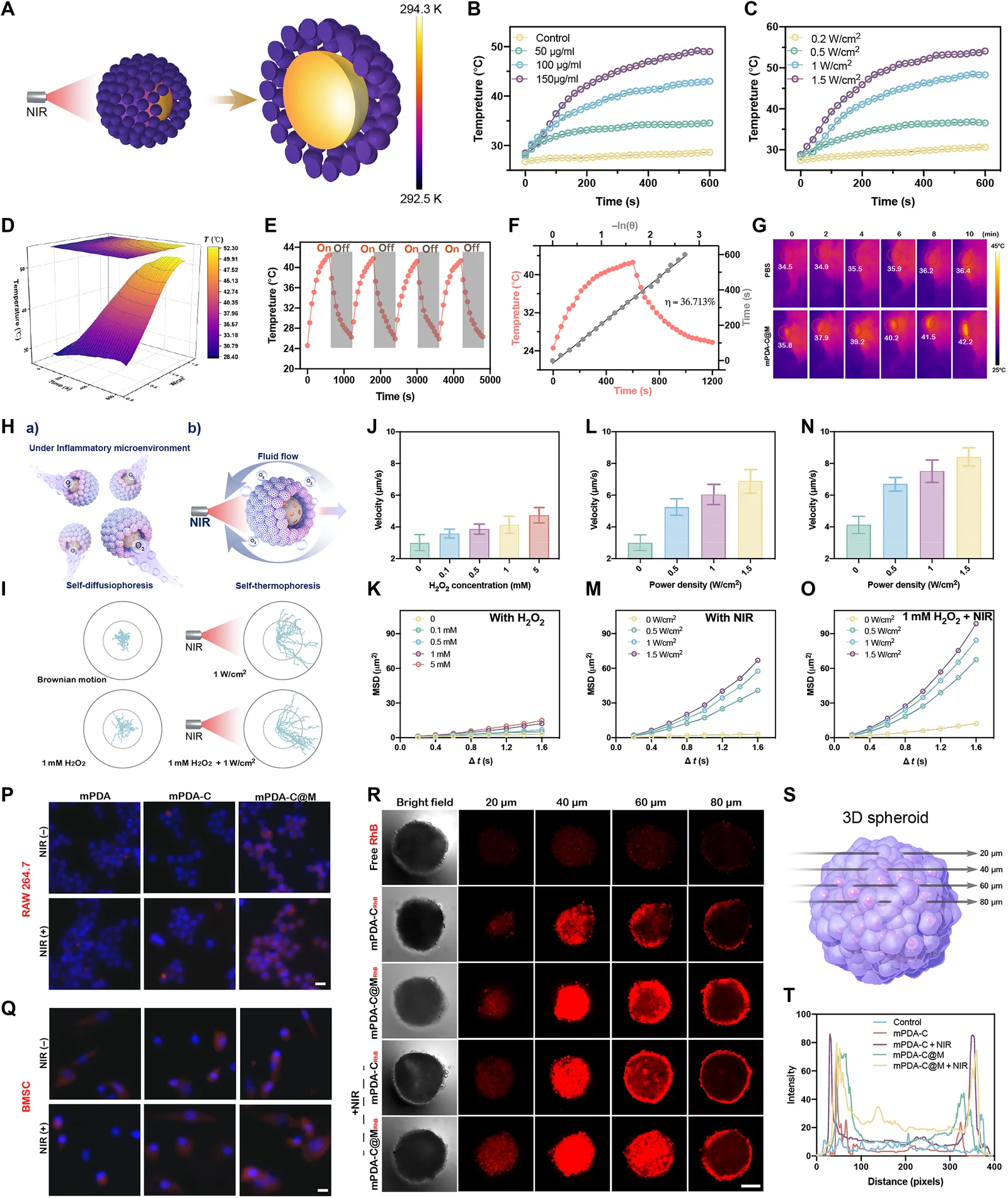
Photothermal performance and motion analysis of mPDA-C@M. (**A**) Schematic illustration of the asymmetric yolk@shell structure of mPDA-C@M and COMSOL-simulated temperature distribution under NIR irradiation. (**B**) Temperature elevation profiles of mPDA-C@M at various concentrations (0, 50, 100, and 150 μg/ml) under NIR irradiation (1.0 W/cm^2^) over 10 min (*n* = 3; mean ± SD). (**C**) Temperature elevation profiles of mPDA-C@M (100 μg/ml) under NIR irradiation at different power densities (0.2, 0.5, 1.0, and 1.5 W/cm^2^) over 10 min (*n* = 3; mean ± SD). (**D**) Plot of maximum temperature change (Δ*T*) versus laser power density. (**E**) Photothermal stability assessment of mPDA-C@M. (**F**) Calculation of the photothermal conversion efficiency (η) of mPDA-C@M (100 μg/ml) under NIR irradiation (1.0 W/cm^2^). (**G**) Representative thermographic images of knee joints treated with PBS or mPDA-C@M. (**H**) Schematic illustration of the dual-propulsion mechanism. (**I**) Average velocities of mPDA-C@M as a function of H_2_O_2_ concentration. (**J**) Average velocities of mPDA-C@M as a function of NIR laser power density. (**K**) Average velocities of mPDA-C@M under different stimulatory conditions (1 mM H_2_O_2_ + NIR). (**L**) Representative motion trajectories of mPDA-C@M under different conditions (*n* = 20) (radius of large circle = 30 μm). (**M**) Mean square displacements (MSDs) of mPDA-C@M as a function of H_2_O_2_ concentration. (**N**) MSDs of mPDA-C@M as a function of NIR laser power density. (**O**) MSDs of mPDA-C@M under different stimulatory conditions. (**P**) Cellular uptake experiments in RAW 264.7 cells and (**Q**) BMSCs. Scale bar, 50 μm. (**R**) Representative confocal fluorescence images showing the permeation of Rhodamine B (RhB)–labeled mPDA-C@M or mPDA-C into BMSC-derived multicellular spheroids (MCSs). Scale bar, 100 μm. (**S**) Schematic illustration of the Z-stack imaging process for MCSs (height ≈ 160 μm) at 20-μm intervals. (**T**) Quantitative fluorescence intensity profiles of nanomotor penetration.

Although PDA is an ideal artificial melanin for photothermal therapy (PTT) (*40*), precise thermal management is critical for cartilage regeneration. Hyperthermia exceeding 45°C impairs chondrocyte metabolism and up-regulates proinflammatory tumor necrosis factor–α (TNF-α) (*41*), while temperatures above 48°C trigger apoptosis and suppress proteoglycan biosynthesis (*31*). Consequently, a mild photothermal temperature of ∼43°C was identified as the optimal therapeutic window to promote tissue repair without cytotoxicity. On the basis of this, 1.0 W/cm^2^ and 100 μg/ml were selected as the standardized parameters for subsequent studies. The photothermal stability of mPDA-C@M was further validated by four consecutive heating/cooling cycles, which showed no discernible attenuation in thermal performance (Fig. 3E). In addition, the photothermal conversion efficiency (η) was calculated to be 36.71% (Fig. 3F), establishing mPDA-C@M as a robust agent for OA MPTT. To further validate this property in vivo, we performed IA injection of mPDA-C@M followed by localized NIR irradiation at the knee joint (Fig. 3G and fig. S4). In contrast to PBS controls, mPDA-C@M–treated joints reached a temperature of ≈42°C, a physiologically relevant threshold conducive to OA PTT. Collectively, these results demonstrate that mPDA-C@M exhibits robust photothermal stability and tunable conversion efficiency while integrating spatiotemporally controlled, heat-mediated therapeutic effects. This unique characteristic establishes mPDA-C@M as a smart nanozyme platform with great potential for precision PTT in OA.

### Motion analysis and enhanced penetration

The motion dynamics of mPDA-C@M were analyzed using optical microscopy, with mean square displacement (MSD) (*42*) and velocity derived from particle trajectories. Capitalizing on its asymmetric yolk-shell architecture, the mPDA-C@M is engineered to harness two synergistic propulsion mechanisms (Fig. 3H). First, the CAT-like activity of the CeO_2_ decomposes H_2_O_2_ to generate an O_2_ gradient, propelling the nanomotor via self-diffusiophoresis driven by osmotic pressure. Given that H_2_O_2_ concentrations in OA joints typically fluctuate (0.1 to 1 mM) (*38*), we evaluated motion across a 0 to 5 mM range. mPDA-C@M displayed concentration-dependent propulsion, with average velocities increasing from 2.9 μm/s (0 mM) to 4.1 μm/s (1 mM) and peaking at 4.7 μm/s (5 mM) (Fig. 3, I to K, and movies S1 and S2). This result indicates that mPDA-C@M exhibits stimulus-responsive mobility beyond passive Brownian diffusion, enabling enhanced diffusion even under low-fuel conditions.

However, while endogenous H_2_O_2_-driven motors show promise, their efficacy is often constrained by the fluctuating and relatively low fuel concentrations in OA joints, which frequently results in erratic propulsion insufficient for deep-tissue infiltration. To overcome this limitation, we further introduced NIR irradiation as an external stimulus to boost nanomotor diffusivity and transport efficiency. The asymmetric shell enables anisotropic absorption of NIR light, creating a local thermal gradient that drives motion via self-thermophoresis (Fig. 3H). NIR-driven nanomotors exhibited higher velocities compared to H_2_O_2_-driven ones, with speed and MSD positively correlating with laser intensity (Fig. 3, L and M, and movie S3). As shown in Fig. 3 (N and O) and movie S4, the dual-driven mode yielded a “turbocharged” effect: The thermophoretic force constructively coupled with the chemical propulsion, markedly amplifying the motion velocity and directionality compared to single-stimulus modes. To quantitatively elucidate the propulsion efficiency, we further compared the kinetic parameters across different driving modes. As shown in Fig. 3I and fig. S5, the dual-driven mode consistently exhibited the highest motility among the tested groups, further supporting the synergistic enhancement produced by coupling chemical propulsion with NIR-triggered thermophoresis.

To evaluate the biological relevance of diffusion-enhanced motion, we examined cellular uptake in RAW 264.7 cells and bone marrow mesenchymal stem cells (BMSCs) under simulated inflammatory conditions in the presence of H_2_O_2_, with or without NIR irradiation. Compared with mPDA and mPDA-C, mPDA-C@M exhibited stronger intracellular fluorescence, indicating that stimulus-enhanced diffusion facilitated cellular internalization (Fig. 3, P and Q, and fig. S6). Notably, NIR irradiation further increased uptake efficiency, and the dual-stimulated group showed the highest fluorescence intensity. Last, to assess the capacity to breach the physical ECM barrier, we used three-dimensional (3D) BMSC-derived multicellular spheroids (MCSs) as a cartilage mimic (Fig. 3, R and S). Representative fluorescence images showed that nonmotile mPDA-C was mainly localized near the spheroid periphery, whereas mPDA-C@M under dual stimulation penetrated more deeply into the spheroid interior. Quantitative fluorescence line-scan analysis across the spheroid diameter further confirmed this trend (Fig. 3T). In particular, the mean fluorescence intensity in the central region of the dual-driven group showed higher central fluorescence intensity than that in the corresponding single-driven group, demonstrating substantially improved penetration into the dense ECM-like structure. Collectively, these findings suggest that the dual-engine strategy effectively converts passive diffusion into stimulus-enhanced diffusion, thereby improving deep-tissue accumulation within dense inflammatory matrices before synovial clearance occurs.

### In vitro inflammation attenuation effect of mPDA-C@M

The avascular nature of articular cartilage severely limits its intrinsic regenerative capacity following injury. In regenerative medicine strategies, BMSCs are prioritized for their chondrogenic differentiation potential and ability to secrete trophic factors that maintain cartilage homeostasis (*31*, *43*). Conversely, RAW 264.7 macrophages, which secrete proinflammatory cytokines and mediate immune responses, were used to recapitulate the inflammatory microenvironment characteristic of OA (*32*, *44*). Therefore, these two cell lines were selected to comprehensively assess the biocompatibility of mPDA-C@M. Initially, rat-derived BMSCs were isolated and characterized at passage 3. Flow cytometric analysis confirmed high purity and phenotypic consistency, with cells expressing surface markers CD90 and CD44 while lacking CD45 (fig. S7). Subsequently, CCK-8 assays demonstrated that both BMSCs and RAW 264.7 macrophages maintained high viability when incubated with mPDA-C@M, comparable to untreated controls, regardless of NIR exposure (figs. S8A and S9A). Consistent with these quantitative findings, live/dead staining visualized predominant cell survival with normal spreading in all groups (figs. S8B and S9B). Although occasional dead cells were noted in NIR-treated RAW 264.7 cultures, the absence of measurable viability reduction indicated negligible cytotoxicity. Collectively, these results confirm the excellent biocompatibility of mPDA-C@M with both stem cells and macrophages, validating its safety profile for subsequent therapeutic applications.

Building on this safety profile, we evaluated the therapeutic efficacy of mPDA-C@M in a coculture model designed to mimic the OA inflammatory microenvironment (*31*). In this system, lipopolysaccharide (LPS)–activated RAW 264.7 macrophages release cytotoxic cytokines and ROS, creating a hostile environment that impairs resident cells. As shown in Fig. 4A, macrophage-induced stress markedly reduced the viability of cocultured BMSCs (68.2%) and chondrocytes (70.6%). However, the introduction of mPDA-C@M markedly restored cell survival, an effect further amplified by NIR irradiation (BMSCs: 89.3%; chondrocytes: 91.8%). This synergistic cytoprotection is driven by three key mechanisms: (i) The mild photothermal effect enhances the kinetic diffusion of mPDA-C@M, maximizing its contact with target cells; (ii) its multienzyme-mimetic activity efficiently scavenges macrophage-derived oxidative stress; (iii) in situ O_2_ generation alleviates inflammation-induced hypoxia, thereby restoring cellular metabolic function. To mechanistically dissect this antioxidative potency, we visualized intracellular ROS levels using DCFH-DA staining. As anticipated, LPS-stimulated macrophages exhibited intense fluorescence, indicative of severe oxidative stress (Fig. 4B). Crucially, the dual-driven motility of mPDA-C@M enables active transport across diffusion barriers, facilitating superior intracellular and intercellular ROS scavenging compared to passive NPs. Consequently, the mPDA-C@M + NIR group achieved the most apparent reduction in ROS, restoring levels to near-normal baselines. Specific probes for O_2_^•−^ (DHE) and hypoxia [Ru(dpp)_3_Cl_2_] further corroborated that mPDA-C@M exerts potent superoxide scavenging and efficient O_2_ generation (Fig. 4C and fig. S10), with its enhanced mobility promoting uniform distribution even in hypoxic, ROS-rich regions. Given that mitochondrial dysfunction in inflammation is perpetuated by a vicious cycle of ROS overload and hypoxia, we further assessed mitochondrial health using JC-1 staining (Fig. 4D and fig. S11). LPS-induced cells displayed a collapsed mitochondrial membrane potential (ΔΨm), evidenced by dominant green fluorescence (JC-1 monomers). In contrast, treatment with mPDA-C@M, particularly under NIR irradiation, effectively reversed this depolarization, yielding a high red-to-green fluorescence ratio comparable to normal cells. This confirms that the nanomotor’s active motion facilitates mitochondrial enrichment, allowing it to precisely target and repair damaged mitochondria by simultaneously neutralizing ROS and supplying O_2_.

**Fig. 4.**
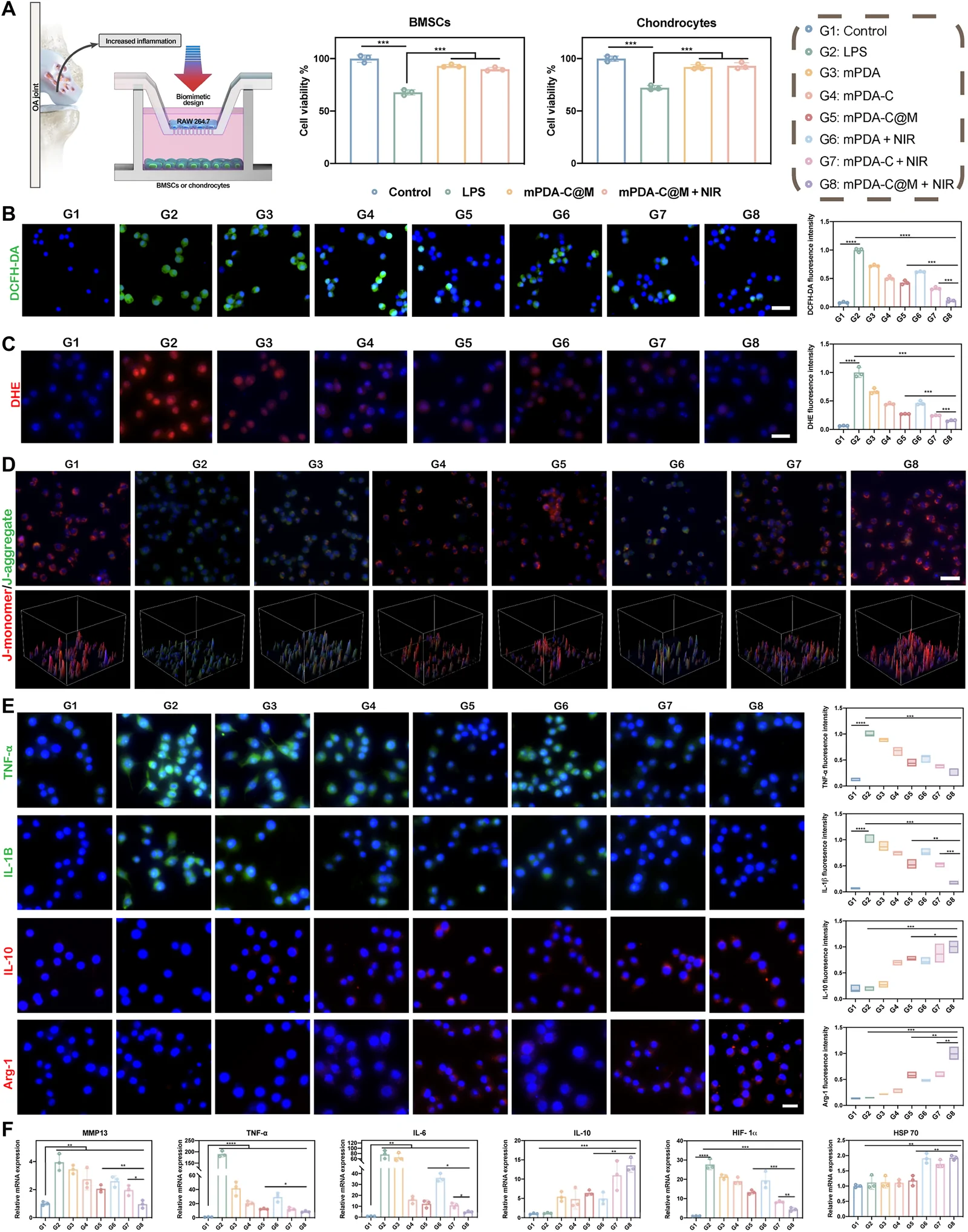
In vitro biocompatibility and anti-inflammatory efficacy of mPDA-C@M. (**A**) Viability of BMSCs and chondrocytes in a coculture system with LPS-induced RAW 264.7 macrophages after different treatments. (**B**) Fluorescence imaging of intracellular ROS levels in LPS-induced RAW 264.7 cells. Scale bar, 50 μm. (**C**) Detection of O_2_^·−^ in LPS-induced RAW 264.7 cells using DHE staining. Scale bar, 50 μm. (**D**) Assessment of mitochondrial membrane potential (ΔΨm) in LPS-induced RAW 264.7 cells via JC-1 staining (red = JC-1 aggregates, indicating intact ΔΨm; green = JC-1 monomers, indicating depolarized ΔΨm). Scale bar, 50 μm. (**E**) Immunofluorescence staining of M1 markers (TNF-α and IL-1β; green) and M2 markers [arginase-1 (Arg-1) and IL-10; red] in LPS-induced RAW 264.7 cells. Scale bar, 50 μm. (**F**) Relative mRNA expression levels of *MMP13*, *TNF-*α, *IL-6*, *IL-10*, *HIF-1*α, and *HSP70* in LPS-induced RAW 264.7 cells. *n* = 3; data are presented as means ± SD. *P < 0.05, **P < 0.01, ***P < 0.001, and ****P < 0.0001.

Reprogramming macrophages from a proinflammatory M1 phenotype to an anti-inflammatory M2 phenotype is a pivotal step in OA resolution (*6*). We evaluated this immunomodulatory effect using immunofluorescence, enzyme-linked immunosorbent assay (ELISA), and quantitative reverse transcription polymerase chain reaction (qRT-PCR). As shown in Fig. 4E, LPS induction polarized macrophages toward the M1 state. While static mPDA and mPDA-C provided partial relief, the dual-driven mPDA-C@M exerted the most profound regulatory effect, shifting the M2/M1 ratio toward that of normal physiological conditions. ELISA results (fig. S12) mirrored this trend, showing maximal suppression of proinflammatory cytokines and up-regulation of anti-inflammatory interleukin-10 (IL-10) in the mPDA-C@M + NIR group. Transcriptional analysis provided further molecular insights (Fig. 4F and fig. S13). Consistent with the attenuation of inflammation, the expression of the key cartilage-degrading enzyme MMP13 was significantly down-regulated in the mPDA-C@M + NIR group, following a trend almost identical to that of M1-related proinflammatory genes (CD86, IL-6, and TNF-α). Conversely, M2 markers (CD163 and IL-10) were markedly up-regulated. Mechanistically, this phenotypic shift was accompanied by the suppression of hypoxia-inducible factor 1α (HIF-1α) and a robust up-regulation of HSP70. Given that HSP70 is known to inhibit inflammatory signaling pathways (e.g., NF-κB), its elevation likely contributes directly to the observed down-regulation of proinflammatory cytokines and MMPs. In conclusion, the dual-driven motion of mPDA-C@M synergistically enhances nanozyme activity and photothermal performance, activating cellular heat-shock defense mechanisms to drive efficient M1 → M2 polarization and suppress catabolic protease expression.

### Biological mechanism of mPDA-C@M in inflammation suppression and chondrogenic differentiation

Accumulating evidence indicates that MPTT-induced up-regulation of HSP70 markedly promotes the chondrogenic differentiation of BMSCs (*31*, *45*). To evaluate the intrinsic chondrogenic potential of mPDA-C@M, we performed Alcian blue staining on BMSCs cultured for 7 and 14 days without standard chondrogenic induction supplements (fig. S14). Notably, the mPDA-C@M group exhibited enhanced staining intensity compared to the chondrogenic induction control at both time points. This effect was further amplified in the mPDA-C@M + NIR group, which displayed the most intense staining by day 14. These findings confirm the superior intrinsic chondrogenic capacity of mPDA-C@M, suggesting that its photothermal stimulation optimizes the microenvironment to direct BMSC differentiation more efficiently than conventional biochemical induction.

To elucidate the molecular basis underlying the therapeutic effects of the dual-driven nanomotor system, we performed transcriptomic profiling of LPS-stimulated BMSCs after different treatments (Fig. 5A). Volcano plots revealed extensive transcriptional dysregulation in LPS-stimulated BMSCs relative to control group. Notably, the inflammatory mediator *Il6* was strongly up-regulated by LPS, whereas the chondrogenic regulator *Sox9* and the stress-responsive chaperone gene *Hspa1b* were altered in a direction consistent with inflammatory impairment of cartilage homeostasis. Following mPDA-C@M + NIR treatment, *Il6* expression was markedly reduced, while *Sox9* and *Hspa1b* were elevated relative to the LPS group (Fig. 5B and fig. S15). This pattern was further supported by the heatmap of representative differentially expressed genes (DEGs), which showed that inflammation- and catabolism-related genes, including *Il1b*, *Tnf*, *Mmp13*, *Mmp9*, and *Adamts5*, were strongly up-regulated by LPS stimulation but substantially suppressed after mPDA-C@M + NIR treatment. In contrast, cartilage anabolic and chondrogenic markers, such as *Acan*, *Col2a1*, *Sox9*, *and Hapln1*, which were down-regulated in the inflammatory state, were restored following dual-driven intervention (Fig. 5C). These results indicate that mPDA-C@M + NIR effectively reprograms the inflammatory transcriptomic landscape toward a cartilage-protective phenotype.

**Fig. 5.**
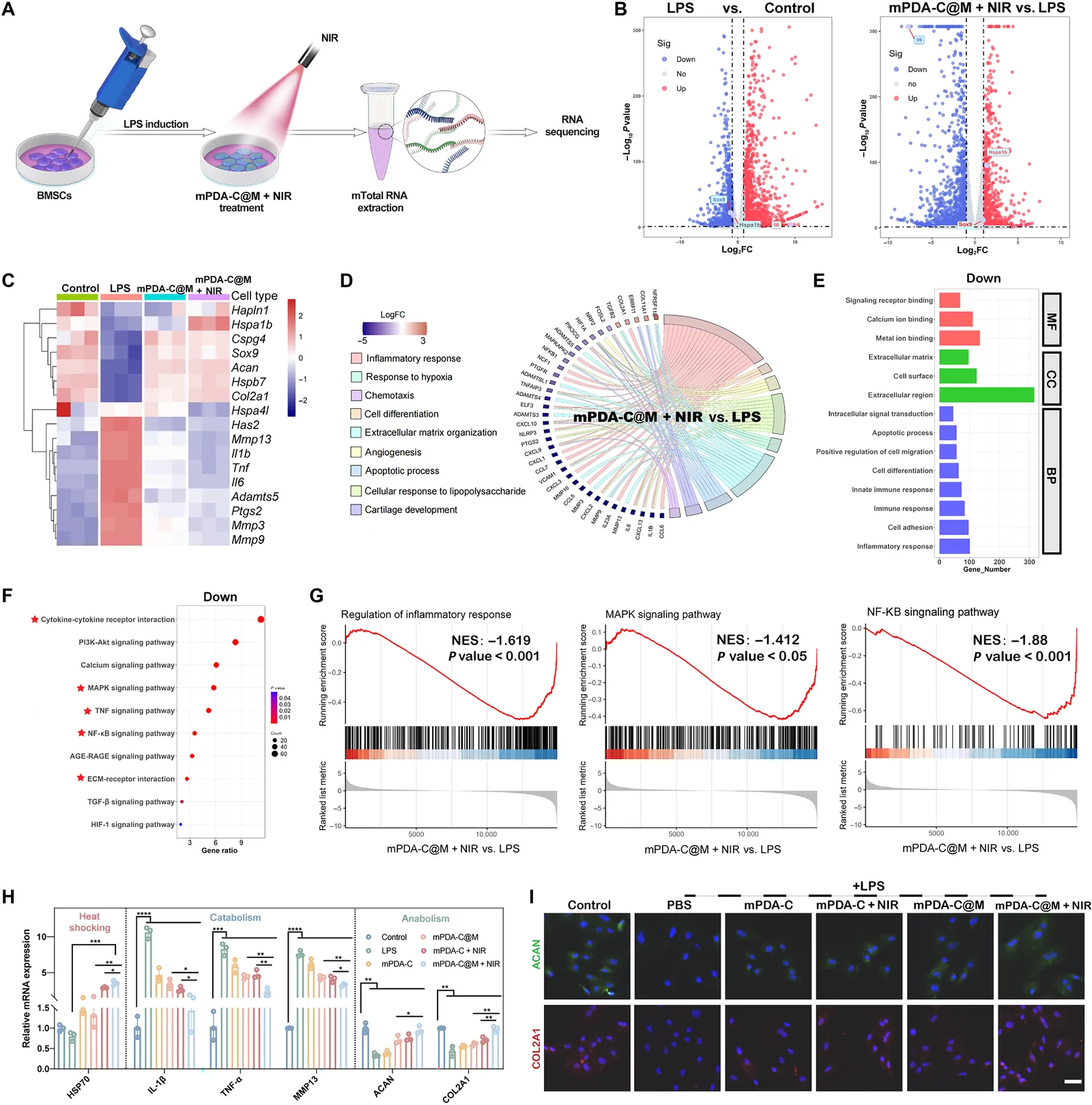
Transcriptomic analysis and molecular mechanisms of mPDA-C@M + NIR treatment in inflammation attenuation and chondrogenesis. (**A**) Schematic diagram of the experimental design: LPS-induced BMSCs were treated with mPDA-C@M or mPDA-C@M + NIR, followed by RNA sequencing (RNA-seq) analysis (*n* = 3). (**B**) Volcano plots of DEGs comparing LPS-induced BMSCs versus control (left) and mPDA-C@M + NIR–treated BMSCs versus LPS group (right) (criteria: fold change ≥1 and *P* < 0.05). (**C**) Heatmap of representative DEGs across different treatment groups. The color scale indicates relative expression levels. The full description of these DEGs is listed in table S1. (**D** and **E**) GO enrichment analysis of biological processes for DEGs in mPDA-C@M + NIR–treated BMSCs versus the LPS-induced group. (**F**) KEGG pathway enrichment analysis of down-regulated pathways in mPDA-C@M + NIR–treated BMSCs relative to the LPS group. PI3K, phosphatidylinositol 3-kinase. (**G**) GSEA showing the differential enrichment of gene sets associated with inflammatory response, MAPK signaling, and NF-κB signaling in mPDA-C@M + NIR–treated versus LPS-stimulated BMSCs. NES, normalized enrichment score. (**H**) qRT-PCR validation of the relative mRNA expression levels of *HSP70*, *IL-1*β, *TNF-*α, *MMP13*, *ACAN*, and *COL2A1*. (**I**) Immunofluorescence of ACAN (green) and COL2A1 (red) in BMSCs. Scale bar, 50 μm. *n* = 3; data are presented as means ± SD. *P < 0.05, **P < 0.01, ***P < 0.001, and ****P < 0.0001.

To further define the molecular programs underlying the therapeutic effect, we performed enrichment analysis of the DEGs regulated by mPDA-C@M + NIR. Gene Ontology (GO) analysis showed that the altered genes were mainly associated with inflammatory response, ECM organization, and stress-related biological processes (Fig. 5, D and E, and figs. S16 and S17), indicating that the treatment simultaneously affected inflammatory remodeling and cartilage metabolic homeostasis. Kyoto Encyclopedia of Genes and Genomes (KEGG) pathway analysis further identified significant enrichment in inflammation-related pathways, particularly the mitogen-activated protein kinase (MAPK) and NF-κB signaling pathways (Fig. 5F and fig. S18). In agreement with these findings, gene set enrichment analysis (GSEA) demonstrated negative enrichment of gene sets associated with inflammatory response, MAPK signaling, and NF-κB signaling in the mPDA-C@M + NIR group compared with the LPS group (Fig. 5G and fig. S19), indicating that the dual-driven treatment effectively attenuated proinflammatory signaling cascades activated under OA-like conditions.

We next validated representative genes by qRT-PCR. The mPDA-C@M + NIR group showed the highest *HSP70* mRNA expression among all groups and significantly exceeded the passive mPDA-C + NIR group (Fig. 5H). This difference is consistent with more effective local photothermal stimulation by the active nanomotor system. In parallel, the expression of inflammatory and catabolic markers, including *IL-1*β, *TNF-*α, and *MMP13*, was reduced, whereas the cartilage anabolic markers aggrecan (*ACAN*) and *COL2A1* were increased. Immunofluorescence staining further confirmed increased ACAN and COL2A1 deposition in the mPDA-C@M + NIR group (Fig. 5I and fig. S20). These findings support an association between HSP70 induction, attenuation of inflammatory/catabolic responses, and restoration of cartilage matrix-associated gene expression.

Together, the transcriptomic and validation data indicate that mPDA-C@M + NIR coordinates the regulation of oxidative stress, inflammatory signaling, and cartilage matrix metabolism. The CeO_2_-containing nanoplatform provides enzyme-like antioxidant activity, while mild NIR photothermal stimulation is associated with enhanced HSP70 expression. In combination with the improved transport properties of the dual-driven nanomotor system, these effects may contribute to suppression of the inflammatory and catabolic OA-like microenvironment and support recovery of an anabolic, reparative phenotype in BMSCs.

### mPDA-C@M with NIR irradiation relieves OA pathological process in a monosodium iodoacetate rat model

Building on the robust in vitro efficacy, we investigated the cartilage-protective effects of mPDA-C@M in a monosodium iodoacetate (MIA)–induced OA rat model. We first addressed the challenge of rapid synovial clearance. Cy7-labeled mPDA-C@M was administered via a single IA injection and monitored using an in vivo imaging system (Fig. 6A and fig. S21). While the fluorescence signal of Cy7 formulation was rapidly cleared at day 2, the Cy7-mPDA-C@M group retained a sustained signal up to day 5. Crucially, NIR irradiation notably prolonged this intraarticular retention. This phenomenon is likely attributed to the motion-enhanced deep tissue retention, which allows the nanomotors to physically “anchor” within the dense ECM, effectively escaping rapid synovial washout. We then evaluated the penetration behavior of the formulations in rat articular cartilage. Compared with the nonirradiated or nonmotile controls, the mPDA-C@M + NIR group showed stronger fluorescence signals within deeper cartilage regions (Fig. 6B), indicating enhanced tissue penetration rather than merely prolonged residence at the joint surface. These findings together suggest that mPDA-C@M not only remains in the joint space for an extended period but also interacts more efficiently with the cartilage lesion region.

**Fig. 6.**
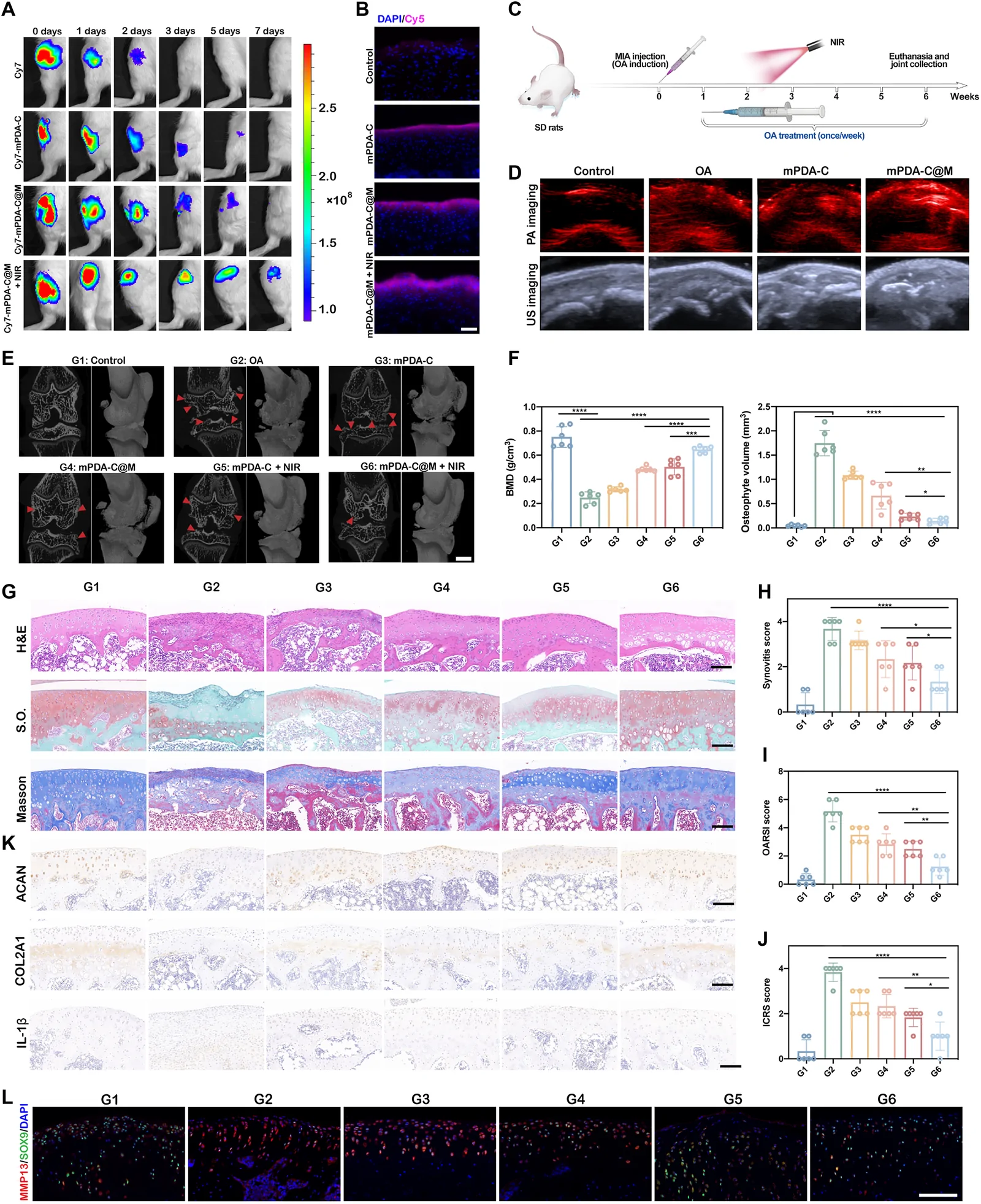
In vivo therapeutic efficacy of mPDA-C@M with NIR irradiation in a MIA-induced OA rat model. (**A**) In vivo fluorescence imaging of rat knee joints following a single IA injection of Cy7-labeled formulations (*n* = 3). (**B**) Representative images of cryosections harvested from MIA-induced rats 24 hours after single IA injection of Cy5-labeled formulations (*n* = 3). (**C**) Schematic timeline of the therapeutic regimen. Male Sprague-Dawley (SD) rats with MIA-induced OA received weekly IA injections of the indicated formulations for 6 weeks (*n* = 6). Created by C.X. using Adobe Illustrator. (**D**) In vivo dual-modal PA/US images of the joint in MIA-induced rats (PA excitation wavelength: 810 nm). (**E**) Representative 2D microcomputed tomography (Micro-CT) coronal sections (left) highlighting osteophytes (red arrows) and subchondral bone structure, alongside 3D reconstructions (right) of the joint surface morphology (*n* = 6). Scale bar, 3 mm. (**F**) Quantitative analysis of bone mineral density (BMD) and total osteophyte volume per joint (*n* = 6). (**G**) Histopathological evaluation of knee joints using hematoxylin and eosin (H&E), Safranin-O/Fast Green (S.O.), and Masson’s trichrome staining (*n* = 6). Scale bars, 50 μm. (**H**) Semiquantitative scoring of synovial inflammation (0 to 4 scale). (**I**) Cartilage degeneration assessed by the OARSI scoring system (*n* = 6). (**J**) Macroscopic cartilage repair evaluated using the ICRS scoring system (*n* = 6). (**K**) Representative immunohistochemical staining and quantitative analysis of COL2A1, ACAN, and IL-1β in articular cartilage sections (*n* = 6). Scale bars, 50 μm. (**L**) Representative immunofluorescence staining of cartilage sections for MMP13 (red) and SOX9 (green), with nuclei counterstained by 4′,6-diamidino-2-phenylindole (DAPI; blue) (*n* = 6). Scale bars, 100 μm. Data are presented as means ± SD.*P < 0.05, **P < 0.01, ***P < 0.001, and ****P < 0.0001.

To evaluate the pathological oxidative microenvironment associated with OA and its relevance to nanomotor propulsion, we quantified H_2_O_2_ content in synovial tissue from rat joints. Compared with the control group, OA joints exhibited a significantly elevated H_2_O_2_ level, confirming the presence of enhanced oxidative stress in the diseased joint microenvironment (fig. S22). Following treatment, the H_2_O_2_ content showed an overall decreasing trend in all intervention groups. Notably, the mPDA-C@M + NIR group displayed the lowest H_2_O_2_ level among the treated groups and showed a significant reduction compared with the OA group. In addition, H_2_O_2_ content in the mPDA-C@M + NIR group was significantly lower than that in the mPDA-C@M group, suggesting that NIR-assisted treatment further strengthened oxidative microenvironment regulation.

Therapeutic efficacy was assessed following a weekly IA injection regimen starting 8 days post–MIA induction (Fig. 6C). Before histological analysis, we evaluated the diagnostic potential of mPDA-C@M (Fig. 6D and fig. S23). In vivo ultrasound imaging revealed distinct echogenic foci within the articular cavity of mPDA-C@M–treated rats (movie S5), substantially exceeding signals in the control and passive mPDA-C groups. This enhancement stems from the vigorous in situ generation of O_2_ microbubbles, catalyzed by the nanomotors specifically in the H_2_O_2_-rich inflammatory microenvironment. Consequently, the signal intensity directly correlates with inflammation severity. Concurrently, mPDA-C@M exhibited superior PA signal intensity, attributed to the synergistic amplification of mPDA’s intrinsic NIR absorption by enhanced motion behavior. This dual-modal imaging capability not only enables the precise visualization of inflammatory lesions but also allows for the real-time monitoring of local nanomotor accumulation, offering a potent theranostic tool for OA management.

OA pathogenesis involves not only cartilage degradation but also severe subchondral bone remodeling (*11*). We used microcomputed tomography (Micro-CT) to assess periarticular bone architecture. Representative 3D reconstructions revealed that the OA model group suffered from severe bone loss and prominent osteophyte protrusions (Fig. 6E), accompanied by significantly reduced bone mineral density (BMD). While mPDA-C@M monotherapy offered partial relief, osteophyte volume remained elevated, indicating insufficient protection against mechanical wear. In stark contrast, the mPDA-C@M + NIR group demonstrated structural preservation: Osteophytes were significantly minimized, and BMD was restored to near-healthy levels (Fig. 6F). Quantitative analysis of tibial subchondral bone microarchitecture [bone surface–to–bone volume ratio (BS/BV), trabecular thickness (Tb.Th), trabecular number (Tb.N), and Tb.Pf] confirmed that the dual-driven therapy effectively normalized trabecular parameters (fig. S24). These results suggest that the synergistic combination of nanomotor-enhanced intraarticular delivery and MPTT effectively halts the “vicious cycle” of joint degradation and aberrant bone remodeling.

To further verify whether these molecular changes could be translated into in vivo therapeutic benefits, we evaluated joint histopathology in the OA model after different treatments. Histological staining, including hematoxylin and eosin (H&E), Safranin O/Fast Green (S.O.), and Masson’s trichrome staining, revealed severe cartilage erosion, proteoglycan loss, and synovial inflammation in the untreated OA group, whereas these pathological changes were substantially alleviated after mPDA-C@M + NIR treatment (Fig. 6G). Quantitative analyses further confirmed that the dual-driven group exhibited the lowest synovial inflammation scores and significantly reduced OARSI scores and Mankin scores, together with the highest ICRS repair scores, among all treatment groups (Fig. 6, H to J, and fig. S25). These results demonstrate that the combination of nanomotor-mediated active penetration and mild photothermal intervention achieves superior joint protection and cartilage repair compared with monotherapy controls.

At the molecular level, immunohistochemical analysis further corroborated the mechanism suggested by the RNA sequencing (RNA-seq) results. OA-induced reductions in the anabolic markers COL2A1 and ACAN, together with the increase in the inflammatory marker IL-1β, were significantly reversed by the dual-driven treatment (Fig. 6K and fig. S26). Notably, the mPDA-C@M + NIR group restored these markers to near-normal levels and consistently outperformed the monotherapy groups. Immunofluorescence staining further showed pronounced up-regulation of SOX9 accompanied by suppression of MMP13 in articular cartilage (Fig. 6L and fig. S27), indicating reactivation of chondrogenic transcriptional programs and inhibition of matrix degradation. These observations support the conclusion that MPTT reestablishes cartilage metabolic balance, while nanomotor-driven penetration helps extend these therapeutic effects into deeper cartilage regions. Consistent with the tissue-level findings, serum ELISA analysis also showed a systemic reduction in inflammatory mediators after mPDA-C@M + NIR treatment (fig. S28). Together, these results demonstrate that the dual-driven nanomotor platform effectively suppresses OA-associated inflammatory signaling, restores anabolic-catabolic homeostasis, and promotes structural and molecular repair of damaged cartilage.

Last, biosafety evaluations confirmed negligible hemolysis and organ toxicity (fig. S29), supporting the clinical translational potential of this platform. Collectively, mPDA-C@M + NIR represents a potent therapeutic strategy that leverages active penetration and photothermal synergy to halt OA progression and promote tissue regeneration.

### mPDA-C@M with NIR irradiation promotes osteochondral regeneration in a cartilage defect rabbit model

To validate the translational potential of the mPDA-C@M system for large-animal cartilage repair, we established a rabbit cartilage defect model. mPDA-C@M was administered via ultrasound-guided in situ injection to ensure precise delivery to the defect site (Fig. 7A). Gross morphological assessment at 6 weeks posttreatment revealed distinct repair outcomes (Fig. 7B). The PBS control group exhibited predominantly fibrous tissue filling with distinct boundaries, indicating poor healing. Similarly, the mPDA-C@M monotherapy group showed limited repair characterized by a rough surface and incomplete integration. In sharp contrast, the mPDA-C@M + NIR group achieved robust tissue regeneration. The defect was filled with smooth, hyaline-like tissue that integrated seamlessly with the surrounding native cartilage, macroscopically resembling the healthy joint surface. Ultrasound imaging further corroborated these findings (Fig. 7C). While the sham group displayed a continuous, smooth hyperechoic line representing the intact articular surface, the mPDA-C@M monotherapy group presented with heterogeneous echogenicity and irregular boundaries, reflecting structural discontinuity. Notably, the mPDA-C@M + NIR group exhibited a smooth, continuous articular contour with echogenic characteristics nearly indistinguishable from the sham group, confirming superior structural reconstruction.

**Fig. 7.**
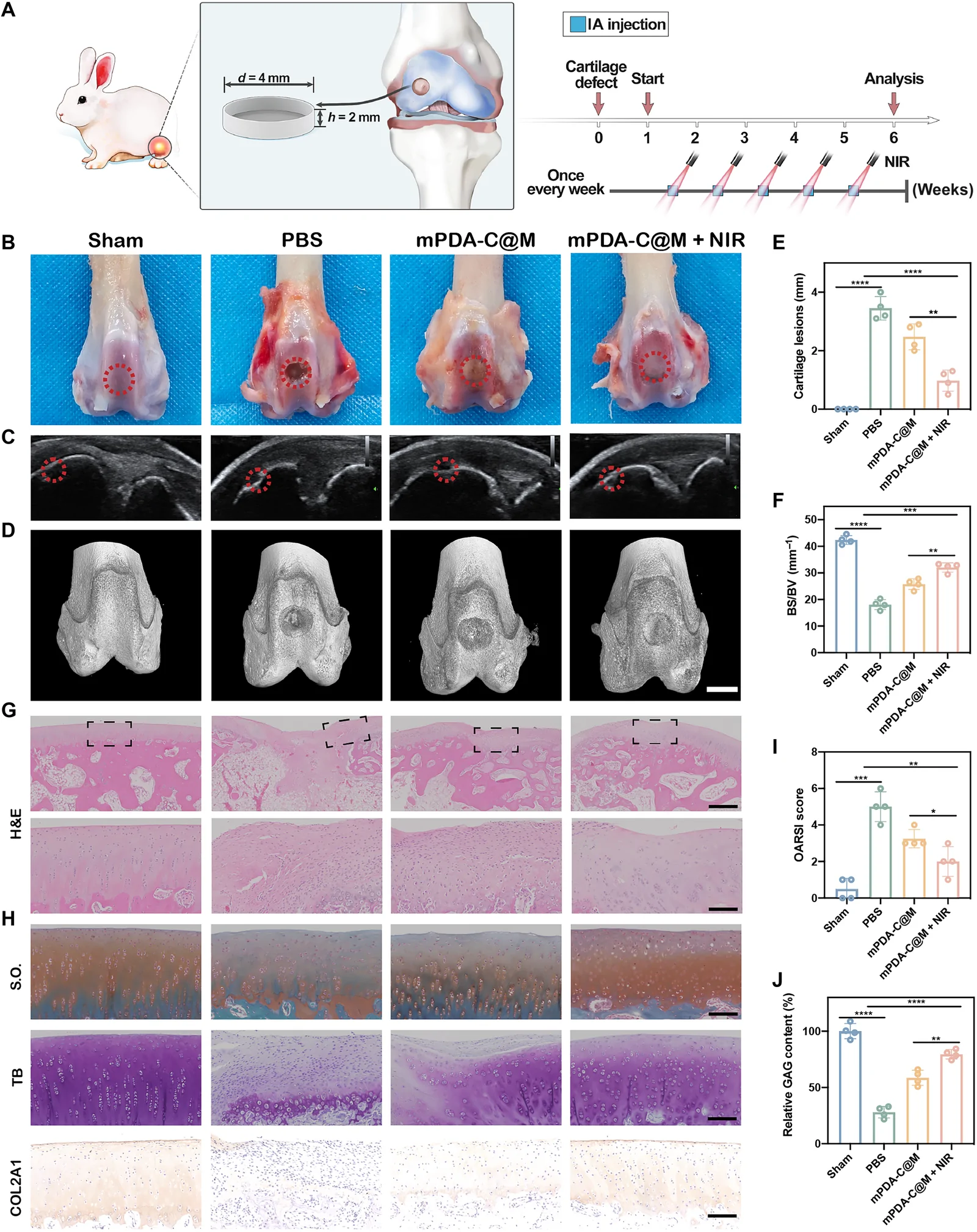
In vivo osteochondral regeneration efficacy of mPDA-C@M under NIR irradiation in a rabbit cartilage defect model. (**A**) Schematic illustration of the surgical induction of cartilage defects in rabbit knee joints, followed by the treatment timeline. (**B**) Representative macroscopic photographs of femoral condyles harvested at 6 weeks postsurgery. (**C**) Representative ultrasound images and (**D**) Micro-CT 3D reconstructions evaluating the structural integrity of cartilage and subchondral bone. Scale bar, 5 mm. (**E**) Quantitative analysis of macroscopic cartilage lesion severity scores. (**F**) Quantitative morphometric analysis of BS/BV in the subchondral bone region. (**G**) Histopathological evaluation of repaired tissues at 6 weeks. Top: H&E staining showing overall tissue morphology (scale bar, 200 μm). Bottom: High-magnification H&E staining (scale bar, 50 μm). (**H**) S.O./Toluidine Blue (TB) staining indicating proteoglycan content, and immunohistochemical staining for COL2A1. Scale bars, 50 μm. (**I**) Histological assessment using the OARSI scoring system. (**J**) Quantitative analysis of relative GAG content based on histological staining intensity. *n* = 4; data are presented as means ± SD. *P < 0.05, **P < 0.01, ***P < 0.001, and ****P < 0.0001.

Given that osteochondral defects involve both cartilage and underlying bone, we assessed subchondral bone remodeling via Micro-CT. At 6 weeks, the PBS group displayed persistent subchondral bone voids and cortical sclerosis at the defect margins. While mPDA-C@M monotherapy offered partial mitigation, the mPDA-C@M + NIR group demonstrated complete osseous bridging and trabecular reconstruction, with no discernible boundary between the regenerated and host bone (Fig. 7D). Quantitative morphometric analysis validated these observations (Fig. 7, E and F). All treatment groups showed improved bone microarchitecture compared to controls. However, the mPDA-C@M + NIR group elicited the most profound recovery. Specifically, BS/BV increased by 77.1% in the dual-driven group (compared to 42.5% in the monotherapy group), accompanied by a significant restoration of BMD (fig. S30). These data underscore that the mild photothermal effect acts synergistically with the nanomotor to accelerate subchondral bone mineralization and remodeling in the rabbit osteochondral defect model.

Histological analysis via H&E, S.O., and Toluidine Blue (TB) staining provided cellular-level insights into the quality of regeneration (Fig. 7, G and H). In the PBS and mPDA-C@M monotherapy groups, the defects were primarily filled with fibrous tissue or fibrocartilage, characterized by weak proteoglycan staining and disorganized cell arrangement. Conversely, the mPDA-C@M + NIR group exhibited high-quality hyaline cartilage regeneration. The repaired tissue displayed intense, uniform staining for S.O. and TB, which is indicative of abundant GAG deposition and exhibited a columnar chondrocyte arrangement that closely mirrored the organization of native cartilage. Furthermore, immunohistochemical staining for COL2A1 revealed robust and uniform expression throughout the matrix in the mPDA-C@M + NIR group. In contrast, the monotherapy group showed weak positivity, and the PBS group was negligible, confirming the phenotype-specific regenerative capacity of the dual-driven therapy. Quantitative assessment using the OARSI scoring system and relative GAG content analysis (Fig. 7, I and J) confirmed statistically significant improvements in the mPDA-C@M + NIR group across all metrics. Collectively, these findings demonstrate that mPDA-C@M under NIR irradiation effectively orchestrates the regeneration of high-quality osteochondral tissue by combining deep ECM penetration with photothermally enhanced metabolic activity.

## DISCUSSION

OA remains difficult to treat in a disease-modifying manner because agents injected into the joint rarely penetrate the deep cartilage regions where inflammatory and catabolic processes persist (*14*, *46*). This is largely due to the dense and avascular cartilage ECM, which limits the diffusion and retention of conventional drugs and passive nanocarriers (*6*). In this study, we developed a dual-energy–driven asymmetric yolk-shell nanomotor, mPDA-C@M, which combines H_2_O_2_-responsive catalytic propulsion with NIR-triggered thermophoretic motion. Within this design, the catalytic module primarily supports autonomous motion in the oxidative OA microenvironment, whereas the NIR-responsive module provides externally controlled motion enhancement together with mild photothermal activation. This cooperative design improved intracartilage transport, increased joint retention, and enhanced accumulation in deep lesion regions, leading to therapeutic benefits in both rat and rabbit OA models.

A key finding is that active transport is central to effective delivery in dense cartilage tissue. Many intraarticular systems can extend residence time in the joint, but after injection, they still rely mainly on passive diffusion and therefore penetrate poorly beyond the cartilage surface (*31*, *47*, *48*). This is a major limitation in OA, where lesions often involve deeper cartilage layers and the osteochondral interface. In our study, mPDA-C@M showed substantially improved movement across cartilage-mimetic barriers and into diseased cartilage. The dual-driven mode also appeared more robust than single-mode propulsion in the heterogeneous joint environment, where endogenous H_2_O_2_ levels may vary with disease stage and local pathology. The asymmetric yolk-shell structure was important for this behavior, as it enabled coordinated catalytic activity and localized photothermal gradient generation under NIR irradiation. Together, these features support the use of combined internal and external energy inputs to improve transport in biologically restricted tissues.

In addition to enhancing delivery under NIR irradiation, mPDA-C@M also actively modulated the osteoarthritic microenvironment. OA progression is associated with oxidative stress, synovial inflammation, macrophage dysregulation, and loss of matrix homeostasis. In this setting, the CeO_2_ component likely reduced oxidative burden, while mild photothermal stimulation under NIR may have further served a dual purpose in this platform, bridging physical transport and biological regulation. Physically, the photothermal conversion of mPDA generates a localized thermal gradient, which synergizes with the catalytic engine to augment thermophoretic propulsion, effectively propelling the nanomotors through the viscous synovial environment. Biologically, the mild local hyperthermia acts as a therapeutic stimulus. It activates cellular protective pathways, evidenced by the elevated expression of HSP70, which helps chondrocytes resist apoptosis under inflammatory stress. Thus, the NIR module functions not merely as a mechanical trigger for motion but as an integral component of the multimodal therapeutic mechanism. The therapeutic benefit of mPDA-C@M therefore appears to arise from the combination of NIR-enhanced tissue access and direct regulation of inflammatory and oxidative pathways by the multifunctional nanomotor system.

The consistent efficacy observed in both rat and rabbit OA models supports the translational potential of this strategy, particularly because joint size, cartilage thickness, and synovial dynamics can strongly influence intraarticular delivery. At the same time, several limitations remain. NIR irradiation may be less efficient in deeper or larger human joints than in small-animal models, and optimization of light delivery will likely be needed. In addition, although short-term biocompatibility was favorable, the long-term fate, biodegradation, and possible off-target accumulation of the nanomotor components require further study, especially in large-animal settings. It will also be important to determine whether this platform remains effective across different stages of OA and under repeated dosing conditions. Overall, our findings suggest that a dual-engine nanomotor can help address transport barriers imposed by the cartilage ECM and improve joint repair in vivo, thereby providing a promising strategy for OA treatment.

## MATERIALS AND METHODS

### Study design

The primary objective of this study was to engineer and validate a dual-driven nanomotor system (mPDA-C@M) capable of overcoming the dense, avascular ECM barrier of articular cartilage to achieve deep tissue penetration and therapeutic efficacy in OA. The study included material synthesis and characterization, evaluation of catalytic, photothermal, and motion properties, ex vivo penetration analysis using 3D chondrogenic spheroids, in vitro assessment of anti-inflammatory and antioxidative effects, and in vivo therapeutic evaluation in OA animal models. For animal experiments, animals were randomly assigned to different treatment groups, and histological analyses were performed in a blinded manner. Detailed group allocation, treatment regimens, and sample sizes are provided in the corresponding sections below.

### Materials and reagents

Chemical reagents were purchased as follows: Cerium(III) acetate sesquihydrate (99%), oleylamine (70%), tetraethyl orthosilicate (TEOS; ≥99%), triethanolamine (TEA; ≥99%), dopamine hydrochloride (≥99%), CTAC (≥99%), and dimethyl sulfoxide (≥99%) were purchased from Sigma-Aldrich (St. Louis, MO, USA). 1,1′-carbonyldiimidazole (CDI; ≥97.0%) and 3-dimethylamino-1-propylamine (DMPDA; ≥99%) were obtained from Macklin Biochemical Co. Ltd. (Shanghai, China). mPEG-DSPE (*M*_W_: 2000) was purchased from Yarebio (Shanghai, China). Primary antibodies against IL-10, arginase-1 (Arg-1), TNF-α, IL-1β, ACAN, MMP13, and collagen type II, as well as Alexa Fluor–conjugated secondary antibodies, were purchased from Proteintech (Wuhan, China). Dulbecco’s modified Eagle’s medium (DMEM), α–minimum essential medium (α-MEM), and fetal bovine serum (FBS) were obtained from Gibco (Thermo Fisher Scientific, Waltham, MA, USA). Deionized water was prepared using a Milli-Q purification system (Millipore, Billerica, MA, USA). All other chemicals were of analytical grade and used as received without further purification.

### Isolation and culture of primary cells

This study used BMSCs, primary chondrocytes, and RAW 264.7 macrophages. Primary BMSCs were isolated from the femurs of 7-day-old Sprague-Dawley (SD) rats as previously described (*31*, *49*). Briefly, after euthanasia and disinfection with 75% ethanol, bone marrow was flushed from the femurs and cultured in complete medium in 6-cm dishes. Nonadherent cells were removed after 3 days, and the medium was replaced every 2 days until the cells reached ∼80% confluence. Primary chondrocytes were isolated from rat articular cartilage. Briefly, hyaline cartilage was harvested, washed three times with PBS, and minced into fragments of <1 mm^3^. The tissue fragments were digested with 0.25% trypsin for 30 min, followed by 0.2% collagenase II for 4 hours. The isolated chondrocytes were collected by centrifugation (1000 rpm for 5 min), resuspended in complete medium, and cultured to 80% confluence.

BMSCs and chondrocytes were cultured in α-MEM supplemented with 20% FBS and 1% penicillin-streptomycin. RAW 264.7 macrophages were cultured in DMEM supplemented with 10% FBS and 1% penicillin-streptomycin. All cells were maintained at 37°C in a humidified incubator containing 5% CO_2_. Chondrogenic induction medium (CIM) consisted of complete DMEM supplemented with 0.1 μM dexamethasone, l-ascorbic acid (50 μg/ml), l-proline (40 μg/ml), sodium pyruvate (100 μg/ml), transforming growth factor–β_3_ (TGF-β_3_; 10 ng/ml), and 1% insulin-transferrin-selenium.

### Animal care

Male SD rats (300 ± 20 g) were obtained from the Laboratory Animal Center of Southern Medical University. Male New Zealand white rabbits (4 to 5 months old and 2.5 to 3.0 kg) were purchased from HuaTeng Biomedical Co. Ltd. Rabbits were housed in single cages, the ambient temperature of the animal room was 18° to 22°C, the daily temperature difference was not more than 4°C, the relative humidity was 40 to 70%, and 12 hours of lighting/12 hours of darkness were alternated between light and darkness. The experimental design and protocols involving animals in this study were approved by the Institutional Animal Care and Use Committee of Southern Medical University (Protocol Registry Number: SMUL202603053) and HuaTeng Biomedical Co. Ltd. (Protocol Registry Number: B202509-11) and followed all aspects of the institutional guidelines. Detailed procedures for rat OA model and rabbit cartilage defect model establishment, randomization, treatment administration, and sample collection are described below.

### Synthesis of ceria NPs

CeO_2_ NPs with a size of ∼5 nm were prepared following a literature method (*36*). Briefly, cerium(III) acetate sesquihydrate (1 mmol) and oleylamine (12 mmol) were dissolved in 15 ml of xylene and stirred at room temperature for 2 hours. The mixture was then heated to 90°C and maintained for 3 hours. After cooling to room temperature, 1 ml of deionized water was added, and the resulting NPs were collected, washed repeatedly with ethanol, and redispersed in chloroform.

To improve water dispersibility and biocompatibility, 5 ml of the ceria NP chloroform dispersion was mixed with 5 ml of mPEG-DSPE solution in chloroform (20 mg/ml) under stirring for 10 min. The solvent was removed by rotary evaporation at 60°C under vacuum for 1 hour. After cooling, 5 ml of deionized water was added, and the mixture was sonicated for 15 min to obtain PEGylated ceria NPs. The product was collected by centrifugation at 20,000 rpm for 30 min and redispersed in deionized water.

### Fabrication of mPDA-C@M

#### Synthesis of functionalized mPDA

mPDA cores were synthesized using a surfactant-templated sol-gel method (*50*). Briefly, dopamine hydrochloride (15 mg), Pluronic F-127 (50 mg), and TMB (30 μl) were dispersed in an ethanol/water mixture, and polymerization was initiated by the addition of aqueous ammonia. After the reaction, the obtained mPDA NPs were collected and further functionalized. For amination, mPDA was reacted with CDI (0.2 g) for 24 hours, followed by the addition of DMPDA (0.5 ml) and 3,3′-iminobis(*N*,*N*-dimethylaminopropyl)amine (1.1 ml), and the reaction was continued for another 24 hours. The product was collected by centrifugation (12,000 rpm for 10 min), washed three times with deionized water, and freeze-dried.

#### Preparation of mPDA-C

PEGylated ceria NPs (60 mg) were mixed with aminated mPDA (20 mg) and stirred overnight to allow surface assembly. The resulting mPDA-C NPs were collected by centrifugation and washed to remove unbound ceria NPs.

#### Preparation of mPDA-C@M

mPDA-C (50 mg) was dispersed in an aqueous solution containing TEA (52 mg) and CTAC (0.2 ml, 20 wt %). TEOS (0.15 ml) was then added dropwise at 70°C, and the reaction was allowed to proceed for 12 hours. The CTAC template was subsequently removed by extraction in acidic ethanol. The resulting mPDA-C@M nanomotors were collected and lyophilized for storage.

### Preparation of fluorescent-labeled NPs

Rhodamine B (RhB)–labeled mPDA, mPDA-C, and mPDA-C@M were prepared by incubating the corresponding NPs with RhB solution under stirring overnight in the dark. The labeled NPs were then collected by centrifugation and washed several times with deionized water to remove free dye. Cy5- and Cy7-labeled mPDA-C and mPDA-C@M were prepared using the same procedure as described for RhB labeling.

### Characterization of mPDA-C@M

The morphology of the NPs was examined by TEM (JEM-1400, JEOL, Japan) operated at 120 kV. SEM and energy-dispersive x-ray spectroscopy were performed using a Phenom SEM system. The hydrodynamic diameter, PDI, and zeta potential were measured by dynamic light scattering (Malvern Instruments, UK). FTIR spectra were recorded using a Nicolet 380 FT-IR spectrometer (Thermo Fisher Scientific, USA). Thermal images were obtained using an infrared thermal camera (FLIR, USA). Heating curves were recorded using an infrared thermal imaging system (Yoseen Infrared, China). The motion behavior of mPDA-C@M was observed under an inverted microscope (Nikon, Japan). The cerium content was determined by inductively coupled plasma mass spectrometry.

### ROS scavenging activity assay

ROS-scavenging activities of mPDA-C@M, mPDA-C, and mPDA against H_2_O_2_, O_2_^•−^, and ·OH were assessed using samples containing an equivalent dosage of NPs. For H_2_O_2_ scavenging, the NPs were incubated with 100 μM H_2_O_2_ for 6 hours, and the residual H_2_O_2_ was quantified using a Hydrogen Peroxide Assay Kit (Solarbio, Beijing, China) according to the manufacturer’s instructions. The H_2_O_2_-scavenging percentage was calculated relative to the H_2_O_2_-only control. Superoxide scavenging activity was assessed using a Superoxide Dismutase (SOD) Assay Kit (Sigma-Aldrich, USA), and ·OH scavenging activity was evaluated using a Hydroxyl Radical Antioxidant Capacity (HORAC) Assay Kit (Cell Biolabs Inc., USA). Unless otherwise specified, all assays were performed according to the manufacturers’ protocols.

### O_2_ generation assay

At room temperature, the O_2_ evolution efficiency of mPDA-C@M, mPDA, and mPDA-C in the presence of 10 mM H_2_O_2_ was measured using an oxygen meter. H_2_O_2_ solution without NPs was used as the background control. Dissolved oxygen was monitored for 15 min, and the results are presented as dissolved O_2_ concentration (in milligrams per liter) as a function of time. In addition, we assessed the O_2_ evolution efficiency of mPDA-C@M at various concentrations.

### Ultrasonographic imaging and PA imaging

In vitro ultrasound imaging was conducted in B-mode using an ACUSON Sequoia system (Siemens, Germany) equipped with an 18-MHz transducer. To simulate the inflammatory environment of arthritis, a custom-made water sac phantom was used. Different concentrations of H_2_O_2_ solution (0.1 and 1 mM) were introduced into the phantom, and the mPDA-C@M suspension was injected via a three-port valve. Changes in echogenic signals were recorded in real time, and the mean gray values of the acquired ultrasound images were quantified using the ImageJ software.

The photoacoustic imaging capability of mPDA-C@M was also evaluated. mPDA-C@M dispersions at concentrations of 0, 25, 50, 100, and 200 μg/ml in PBS were loaded into capillary tubes and imaged using a Vevo LAZR-X PA imaging system (VisualSonics Inc., Canada). Image processing and quantitative analysis were performed using the Vevo LAZR software package.

### Photothermal properties

mPDA-C@M suspensions at varying concentrations (0 to 150 μg/ml) were irradiated with an 808-nm NIR laser at 1.0 W/cm^2^ for 10 min. To assess power-dependent heating, a fixed concentration of mPDA-C@M (100 μg·ml^−1^) was exposed to NIR light at 0.5 to 1.5 W/cm^2^ for 10 min, and the temperature changes were recorded over time. For concentration-dependent photothermal tests, mPDA-C@M samples (0 to 150 μg/ml) were placed in 2-ml EP tubes, with PBS serving as a control. All samples were irradiated at 1 W/cm^2^ for 10 min, and infrared thermal images were captured to visualize the temperature distribution. The photostability of mPDA-C@M was evaluated by subjecting the suspension (100 μg/ml) to four repeated heating-cooling cycles under laser irradiation (1.0 W/cm^2^).

### Photothermal conversion efficiency

An mPDA-C@M suspension (100 μg/ml) was placed in a 2-ml microcentrifuge tube and irradiated with an 808-nm laser at 1.0 W/cm^2^ for 600 s. The temperature variation during laser heating and natural cooling was recorded, and the photothermal conversion efficiency (η) was calculated according to a previously reported method (*23*). Briefly, the heating and cooling profiles of mPDA-C@M were recorded, and η was calculated using the equation

$$\eta =\frac{\mathrm{hS}\left(T_{\text{max}}-T_{\text{RT}}\right)-Q_{\text{dis}}}{I\left(1-10^{-A_{808}}\right)}$$ (1)

where *hS* = thermal transfer coefficient, *T*_max_ = sample equilibrium temperature, *T*_RT_ = room temperature, *Q*_dis_ = system heat loss, *I* = laser power density, and *A*_808_ = absorbance at 808 nm. The *hS* value was derived from

$$\mathrm{hS}=\frac{\mathrm{mc}}{\tau_{s}}$$ (2)

where *m* = sample mass, *c* = specific heat capacity of water, and τ*_s_* = time constant, with τ_s_ computed via

$$T=-\tau_{s}\text{ln}\left(\theta \right)$$ (3)

θ = dimensionless parameter, defined as the temperature difference between the sample and ambient environment

$$\theta =\frac{T-T_{\text{RT}}}{T_{\text{max}}-T_{\text{RT}}}$$ (4)

Accordingly, the photothermal conversion efficiency of mPDA-C@M under 808-nm laser irradiation was 36.71%.

### Motion analysis

The motion behavior of mPDA-C@M nanomotors was recorded using a Nikon Ti2-A inverted microscope equipped with a high-speed sCMOS camera and NIS-Elements AR 3.2 software. The trajectories of the nanomotors were recorded in the presence of different H_2_O_2_ concentrations (0, 0.1, 0.5, 1, and 5 mM), under laser irradiation at different power densities (0.5, 1.0, and 1.5 W/cm^2^), and under the combined condition of 1 mM H_2_O_2_ plus laser irradiation (1.0 W/cm^2^). For each condition, videos were recorded for 10 s, and at least 20 individual particles were analyzed. Particle trajectories were tracked using the Manual Tracking plugin in ImageJ, and the particle velocities were calculated accordingly. MSD values were calculated following a previously reported method (*27*, *42*).

### Enhanced penetration in 3D spheroids

To assess the penetration capability of mPDA-C@M in a cartilage-mimetic ECM environment, a 3D MCS model was established. BMSCs were seeded in U-bottom plates at a density of 5 × 10^4^ cells per well and centrifuged at 250*g* for 5 min. The plates were then incubated at 37°C in 5% CO_2_ for 24 hours to facilitate spheroid formation. The spheroids were subsequently cultured in CIM, which was refreshed every 2 to 3 days, until spheroids with diameters of ∼100 μm were obtained. For penetration studies, the culture medium was replaced with suspensions of RhB-labeled mPDA-C@M or mPDA-C, followed by NIR irradiation where indicated. After incubation, the spheroids were washed with PBS and imaged by confocal laser scanning microscopy. Z-stack images were collected at 10-μm intervals to evaluate penetration depth.

### Cell cytotoxicity

The effects of mPDA-C@M, mPDA, and mPDA-C on the viability of RAW 264.7 macrophages and BMSCs were evaluated using a CCK-8 assay (Solarbio, Beijing, China). Cells were seeded in 96-well plates at a density of 8 × 10^3^ cells per well and cultured for 24 hours. The cells were then treated with mPDA-C@M, mPDA, or mPDA-C. After an additional 12-hour incubation, the cells were exposed to NIR irradiation or left untreated, and cell viability was determined according to the manufacturer’s instructions.

Live/dead staining was also performed using Calcein-AM and propidium iodide (Solarbio, Beijing, China). After 24 hours of incubation with mPDA-C@M, mPDA, or mPDA-C, the cells were stained with the working solution for 20 min and imaged under a fluorescence microscope.

### Cellular uptake

RAW 264.7 macrophages and BMSCs were seeded in 96-well plates at a density of 8 × 10^3^cells per well and cultured for 24 hours before the experiments. The cells were then stimulated with LPS (Solarbio, Beijing, China) (1 μg/ml) for 6 hours. Subsequently, the culture medium was replaced with RhB-labeled mPDA-C@M, mPDA, or mPDA-C. After washing with PBS, the cells were subjected to different conditions in the presence or absence of H_2_O_2_ (1 mM) and NIR irradiation (1.0 W/cm^2^). Following 6 hours of incubation, the cells were washed and stained with Hoechst for 15 min. Cellular uptake was then observed using a fluorescence microscope.

### Coculture system to simulate OA microenvironment

A transwell coculture system was used to evaluate whether the anti-inflammatory effect of mPDA-C@M under NIR irradiation could improve the viability of normal cells in an inflammatory microenvironment (*51*). Briefly, RAW 264.7 macrophages were seeded in transwell inserts at a density of 5000 cells per well and stimulated with LPS for 24 hours to induce inflammation. Meanwhile, BMSCs and chondrocytes were seeded in 24-well plates at a density of 10 000 cells per well. The inserts were then transferred to the corresponding wells, and culture medium containing mPDA-C@M was added. In the mPDA-C@M + NIR group, the cells were exposed to NIR irradiation for 10 min. After 24 hours of coculture, the inserts were removed, and 10 μl of CCK-8 reagent was added to each well. Following incubation for 3 hours, the absorbance at 450 nm was measured using a microplate reader.

### Free radical scavenging

·OH and O_2_^•−^ were selected as indicators to assess the intracellular free radical scavenging capacity of mPDA-C@M. RAW 264.7 cells were seeded in 24-well plates at a density of 2 × 10^4^ cells per well and allowed to adhere. The cells were then stimulated with LPS (1 μg/ml) to induce inflammation. After LPS stimulation, mPDA-C@M was added to the culture medium. Intracellular ·OH and O_2_^·−^ levels were detected using DCFH-DA and DHE fluorescent probes (Solarbio, Beijing, China), respectively. Fluorescence images were acquired using a fluorescence microscope, and fluorescence intensity was quantified with ImageJ.

### Intracellular O_2_ evaluation

To simulate hypoxic conditions in vitro, RAW 264.7 macrophages were cultured under low-oxygen conditions for 24 hours. The cells were then incubated with the [Ru(dpp)_3_]Cl_2_ oxygen probe (Solarbio, Beijing, China) for 2 hours under continued hypoxia to allow intracellular uptake and oxygen-responsive fluorescence. Thereafter, the cells were treated with mPDA-C@M, mPDA, or mPDA-C and maintained under hypoxia for another 8 hours. Subsequently, 100 μM H_2_O_2_ was added to each well and incubated for 1 hour. After washing with PBS to remove excess H_2_O_2_ and uninternalized materials, the cells were subjected to NIR irradiation and further incubated for 2 hours. The fluorescence intensity of [Ru(dpp)_3_]Cl_2_ was then monitored using an inverted fluorescence microscope.

### Mitochondrial membrane potential detection

The mitochondrial membrane potential (ΔΨm) of RAW 264.7 macrophages after different treatments was assessed using a JC-1 assay kit (Solarbio, Beijing, China). Following the indicated treatments, the cells were incubated with JC-1 staining solution for 30 min at 37°C and then gently washed with PBS to remove excess dye. Fluorescence images of JC-1 monomers (green fluorescence) and aggregates (orange-red fluorescence) were acquired using a fluorescence microscope. The ratio of aggregate to monomer fluorescence intensity was quantified using the ImageJ software to evaluate changes in mitochondrial membrane potential.

### Chondrogenic differentiation of BMSC

To evaluate chondrogenic differentiation under an inflammatory microenvironment, BMSCs were seeded in six-well plates at a density of 50,000 cells per well and exposed to LPS (1 μg/ml) for 24 hours. The cells were then divided into the indicated groups and cultured in medium containing mPDA-C@M, with or without NIR irradiation. The medium was replaced every 2 days throughout the 2-week induction period. Cells maintained in standard medium without LPS or material treatment served as the control.

### Alcian blue staining

Alcian blue staining was performed to evaluate chondrogenic differentiation of BMSCs by detecting sulfated GAG deposition. Cells were seeded in culture plates and allowed to adhere for 24 hours. After 1 or 2 weeks of chondrogenic induction, the cultures were washed with PBS, fixed with 4% paraformaldehyde, and rinsed thoroughly with distilled water. The samples were then stained with Alcian Blue solution at room temperature for 30 min, followed by gentle washing to remove excess dye. A counterstain was subsequently applied for 30 to 60 s, after which the samples were rinsed again and air-dried. Staining intensity was evaluated after complete drying.

### RNA-seq and data analysis

Following sequential treatment with LPS, mPDA-C@M, and NIR irradiation, total RNA was extracted from BMSCs using TRIzol reagent (TOYOBO, Japan). RNA-seq libraries were constructed and sequenced on an Illumina NovaSeq 6000 platform (Illumina, USA) by Novogene Biotech Co. Ltd. (Beijing, China). To investigate the underlying biological mechanisms, GO, KEGG pathway enrichment, and GSEA were performed. A corrected *P* value (adjusted *P*) < 0.05 was used as the threshold for identifying significantly DEGs and significantly enriched GO terms and KEGG pathways. The full description of DEGs were listed in table S1.

### Immunofluorescence assay

For BMSCs, cells were seeded on glass slides in 12-well plates at a density of 5000 cells per well and cultured in CIM containing mPDA-C@M. The medium was replaced every 2 days, and NIR irradiation was applied to the corresponding group. After 7 days, the cells were fixed with 4% paraformaldehyde for 30 min. According to standard immunofluorescence procedures, the samples were incubated overnight at 4°C with primary antibodies against ACAN and COL2A1, followed by incubation with the corresponding fluorescent secondary antibodies (goat anti-rabbit IgG–Alexa Fluor 488 and goat anti-mouse IgG–Alexa Fluor 647). Nuclei were counterstained with 4′,6-diamidino-2-phenylindole (DAPI), and fluorescence images were acquired using a fluorescence microscope. The fluorescence intensities of ACAN and COL2A1 were quantified using ImageJ.

For RAW 264.7 macrophages, LPS-stimulated and material-treated cells were fixed with 4% paraformaldehyde, permeabilized, blocked, and subjected to the same immunofluorescence staining procedure as described above, including incubation with primary and secondary antibodies, DAPI counterstaining, and imaging using an inverted fluorescence microscope.

### qRT-PCR analysis

Total RNA from BMSCs and RAW 264.7 macrophages was extracted using a Super Total RNA Isolation Kit (Foregene) according to the manufacturer’s instructions. The isolated RNA was reverse transcribed into cDNA using a cDNA ReverTra Ace qPCR RT Kit (TOYOBO). Quantitative real-time PCR was performed with SYBR Green Realtime PCR Master Mix (TOYOBO) on a LightCycler 480 Instrument (Roche). The PCR program consisted of an initial denaturation at 95°C for 10 min, followed by 40 cycles of denaturation at 95°C for 15 s and annealing/extension at 60°C for 1 min. The primers were presented in tables S2 and S3. Relative mRNA expression levels were calculated using the 2^−ΔΔ*C*t^ method.

### ELISA

To assess the anti-inflammatory effects of the NPs, RAW 264.7 macrophages stimulated with LPS (1 μg/ml) were seeded in 12-well plates and incubated with mPDA-C@M, mPDA, or mPDA-C for 24 hours. The concentrations of inflammatory cytokines in the supernatants were measured using mouse IL-10, IL-1β, and TNF-α ELISA kits (BioLegend, USA), according to the manufacturers’ instructions.

### Animal model induction and treatment

#### Rat OA model

Male SD rats weighing 300 ± 20 g were used to establish the OA model by a single intraarticular injection of MIA (50 μl, 40 mg/ml) into the knee joint. On day 14 after MIA injection, when early inflammatory manifestations were evident, the rats were randomly assigned into six groups, with healthy rats serving as the normal control: (i) normal control, (ii) OA model, (iii) mPDA-C, (iv) mPDA-C@M, (v) mPDA-C + NIR, and (vi) mPDA-C@M + NIR. Each group received a 100-μl intraarticular injection of the corresponding formulation under real-time ultrasound guidance using an 18-MHz transducer. The treatments were administered once weekly. For the NIR groups, the knee joints were irradiated with an 808-nm laser at a power density of 1 W/cm^2^ for 10 min. Thermal images of the knee joints were acquired every 2 min during irradiation using a thermal camera. All rats were euthanized at 6 weeks after treatment, and joint tissues, serum samples, and major organs (heart, liver, spleen, lungs, and kidneys) were collected for subsequent analyses, including imaging, histology, and cytokine measurement.

#### Rabbit cartilage defect model

A rabbit cartilage defect model was established to further evaluate the in vivo cartilage repair performance of the mPDA-C@M system. Muscle anesthesia was processed on each rabbit. Subsequently, under aseptic conditions, an incision was created on the knee from the lateral side, and the joint was exposed following dislocation of the patella. Using a corneal trephine, a cylindrical defect with dimensions of 4 mm in diameter and 2 mm in depth was then fashioned on the trochlear groove of the distal femur. After that, the patella was relocated, and the incision was sutured. Then, 16 rabbits were randomly divided into four groups: sham, PBS, mPDA-C@M, and mPDA-C@M + NIR. In situ injection of mPDA-C@M, or PBS as a control, was performed at the defect site under ultrasound guidance. For the combined treatment group, the defect area was irradiated with an 808-nm NIR laser at 1 W/cm^2^ for 10 min after injection. At 6 weeks posttreatment, the defect-bearing knee joints were harvested for macroscopic observation, Micro-CT analysis, and ultrasound imaging to evaluate subchondral bone regeneration. Histological staining, including H&E, S.O., and TB staining, as well as OARSI scoring, was performed to comprehensively assess cartilage repair outcomes. The number of animals per group was selected on the basis of the 3Rs (replacement, reduction, and refinement) guidelines, ensuring minimal animal use while still achieving sufficient statistical power or meaningful comparisons.

### In vivo biodistribution assay

For in vivo biodistribution analysis, MIA-induced OA rats received an IA injection of Cy7-labeled NPs into the knee joint. In vivo fluorescence imaging was performed at 0, 1, 3, 5, 7, and 10 days postinjection using an IVIS imaging system. The imaging parameters were set as follows: excitation wavelength: 745 nm; emission wavelength: 790 nm; exposure time: 2 s. Images and quantitative fluorescence data were processed using the Living Image software.

### In vivo cartilage penetration

To evaluate cartilage penetration in vivo, OA rats were randomly divided into four groups and intraarticularly injected with Cy5-labeled NPs with or without NIR irradiation. At 24 hours after injection, the rats were euthanized, and the articular cartilage from the knee joints was harvested and washed with PBS. The fresh samples were then immediately embedded in Tissue-Tek optimal cutting temperature compound and cryosectioned into 10-μm slices (Leica Biosystems). The sections were mounted with DAPI and immediately observed under a fluorescence microscope to assess NP distribution and cartilage penetration.

### H_2_O_2_ quantification of synovial tissue

A Hydrogen Peroxide Assay Kit (BestBio, Shanghai, China) was used to quantify the H_2_O_2_ level in the synovial tissue of OA rats, based on the titanium sulfate method, in which H_2_O_2_ reacts with titanium sulfate to form a colored complex with maximum absorbance at 412 nm. For H_2_O_2_ detection, fresh synovial tissues were collected and homogenized in ice-cold saline to prepare homogenates. The homogenates were then centrifuged at 12,000*g* for 10 min, and the supernatants were collected for analysis. The H_2_O_2_ content in the synovial tissue homogenates was subsequently determined by measuring the absorbance at 412 nm using a UV-vis spectrophotometer, according to the manufacturer’s instructions.

### In vivo US and PA imaging

For ultrasound imaging, OA rats were anesthetized and positioned for in vivo joint examination using an ACUSON Sequoia imaging system (Siemens, Germany) equipped with an 18-MHz transducer. mPDA-C@M was injected intraarticularly into the knee joint, and sequential ultrasound scans were acquired before and after injection. Real-time changes in echogenic signals within the joint cavity were recorded, and the mean gray values of the ultrasound images were quantified using the ImageJ software.

For PA imaging, a Vevo LAZR-X PA imaging system (VisualSonics Inc., Canada) equipped with an LZ550 transducer was used. After intraarticular administration of mPDA-C@M, the knee joints of rats were scanned in PA mode. Image acquisition was performed at 810 nm to determine the spatial distribution and retention of the material within the joint. Image reconstruction and data analysis were conducted using the accompanying software.

### Micro-CT analysis

Knee joint specimens were fixed in 4% paraformaldehyde for 3 days and then scanned using a high-resolution Micro-CT system (SkyScan 1176, Bruker, Belgium). 3D reconstruction of the scanned images was performed using standardized parameters. Bone morphometric indices, including osteophyte volume, BMD, BS/BV, Tb.N, and Tb.Th, were quantitatively analyzed using the CTAn software (version 1.13.8.1) based on the reconstructed 2D/3D datasets.

### Histology, immunohistochemistry, and immunofluorescence analyses

Knee joints were collected and processed by fixation, decalcification, dehydration, and paraffin embedding. Histological sections were stained with H&E for general morphology, Masson’s trichrome for collagen deposition, and S.O. for cartilage matrix evaluation. Cartilage degeneration was graded according to the OARSI scoring system based on S.O.-stained sections. Scoring was performed independently by three blinded evaluators.

For immunohistochemistry and immunofluorescence analyses, tissue sections were subjected to heat-induced antigen retrieval, blocked, and incubated overnight at 4°C with the indicated primary antibodies. Subsequent staining procedures were conducted according to standard protocols.

### Quantification of serum cytokines

Serum samples from rats were collected on day 56, and the concentrations of IL-10, IL-6, and TNF-α were quantified by ELISA. Briefly, whole blood was collected by tail cutting and allowed to clot at room temperature for 30 min. The samples were then centrifuged at 4000*g* for 20 min to obtain serum. The serum samples were immediately stored at −20°C and analyzed within 3 days using rat IL-10, rat IL-6, and rat TNF-α ELISA kits (BioLegend, USA).

### Biosafety evaluation

To evaluate the systemic biosafety of the NPs, blood samples and major organs, including the heart, liver, spleen, lungs, and kidneys, were collected for histopathological examination. Samples from healthy rats were used as controls.

For hemolysis analysis, whole blood from normal rats containing EDTA was centrifuged (3000 rpm) for 8 min, and the obtained erythrocytes were washed with PBS. Red blood cell (RBC) solution was 1 ml of PBS resuspension containing 200 μl of erythrocytes. A 20-μl RBC solution was mixed in 980 μl of mPDA-C@M solution (final concentrations of mPDA-C@M were 25 to 200 μg/ml). Negative control group and positive control group were 20 μl of RBC solution mixed with 980 μl of PBS and 980 μl of deionized water during 2 hours of incubation at 37°C. Last, photographs were captured, all samples were centrifuged (3000 rpm), and 100 μl of supernatant was obtained for the measurement of the absorbance at 570 nm. The formula for calculating the percentage of hemolysis was as follows

$$\begin{array}{c} \text{Hemolysis}\;\left(\%\right)=\\ \frac{\left(\text{sample absorbance}-\text{negative control absorbance}\right)}{\left(\text{positive control absorbance}-\text{negative control absorbance}\right)}\times 100 \end{array}$$ (5)

### Statistical analysis

All data are presented as the means ± SD. Biological replicates were used in both in vitro and in vivo experiments. Comparisons between two independent groups were analyzed using an unpaired, two-sided Student’s *t* test; multiple comparisons were conducted using one-way or two-way analysis of variance (ANOVA) with Tukey’s post hoc test. All statistical analyses were performed using GraphPad Prism version 8.0 software. Differences were considered statistically significant at *P < 0.05, **P < 0.01, ***P < 0.001, and ****P < 0.0001.
