# Supplementary material for: NIR-enzymatic dual-engine nanomotors overcome cartilage matrix barriers and reprogram the osteoarthritic microenvironment
Source: Sci Adv. 2026 Nov 13;12(46):eaej0289. doi: 10.1126/sciadv.aej0289 (PMC13763856; doi:10.1126/sciadv.aej0289)
Supplement: Supplementary file 1 [file sciadv.aej0289_sm.pdf]

Science Advances
AAAS

Supplementary Materials for

NIR-enzymatic dual-engine nanomotors overcome cartilage matrix barriers and reprogram the osteoarthritic microenvironment

Cong Xu *et al.*

Corresponding author: Lihan Shen, shenlihan@hotmail.com; Yingfeng Tu, tuyingfeng1@smu.edu.cn; Yingjia Li, lyjia@smu.edu.cn

Sci. Adv. **12**, eaej0289 (2026)
DOI: 10.1126/sciadv.aej0289

The PDF file includes:

Figs. S1 to S30
Tables S1 to S3
Legends for movies S1 to S5

Other Supplementary Material for this manuscript includes the following:

Movies S1 to S5

Supplementary Figures

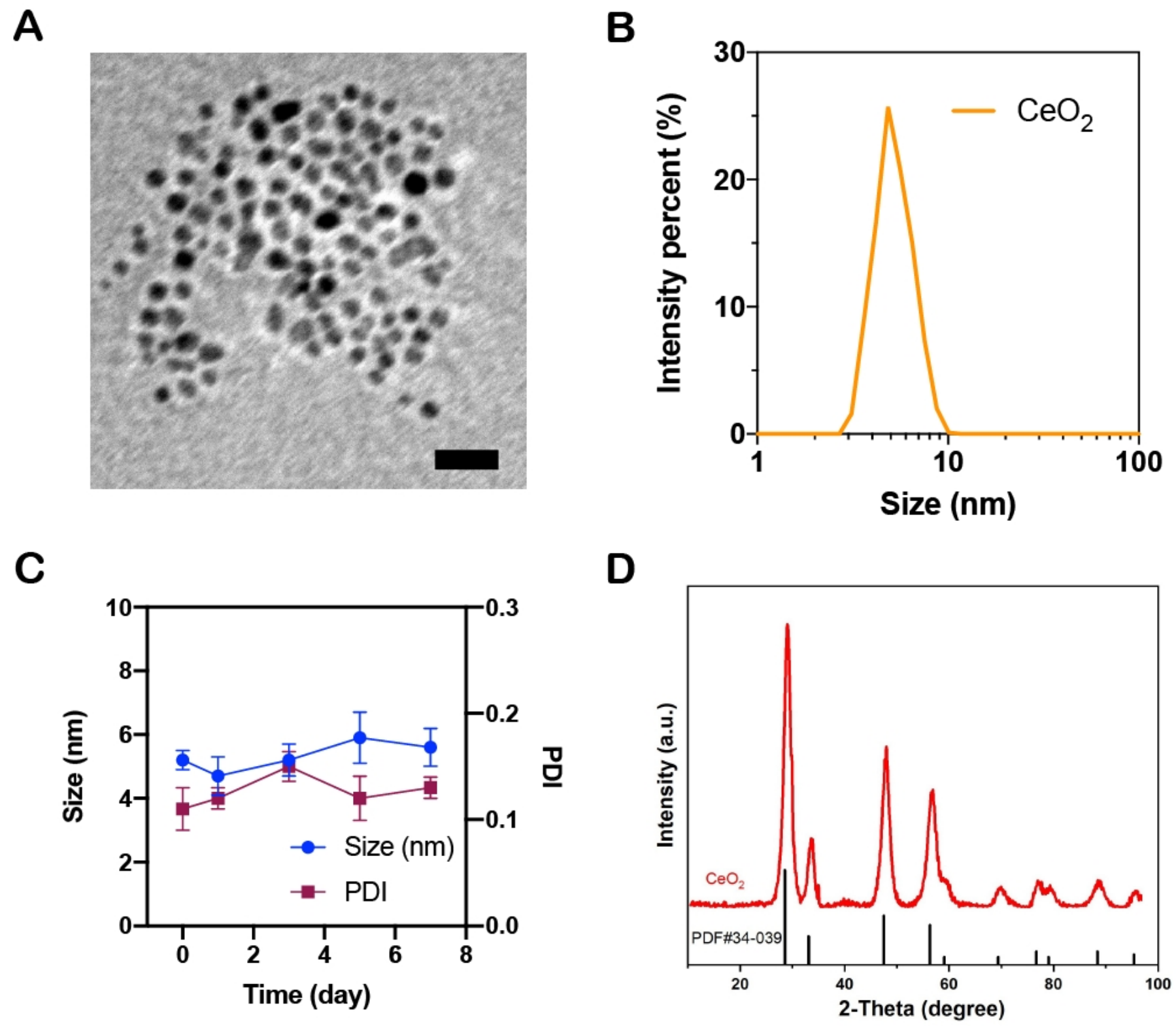

Fig. S1. Characterization of CeO_2 NPs. (A) TEM image of CeO_2 NPs. (B) Size distribution of CeO_2 NPs. (C) Colloidal stability evaluation of CeO_2 NPs in PBS (n = 3, mean ± SD). (D) X-ray powder diffraction of CeO_2 NPs.

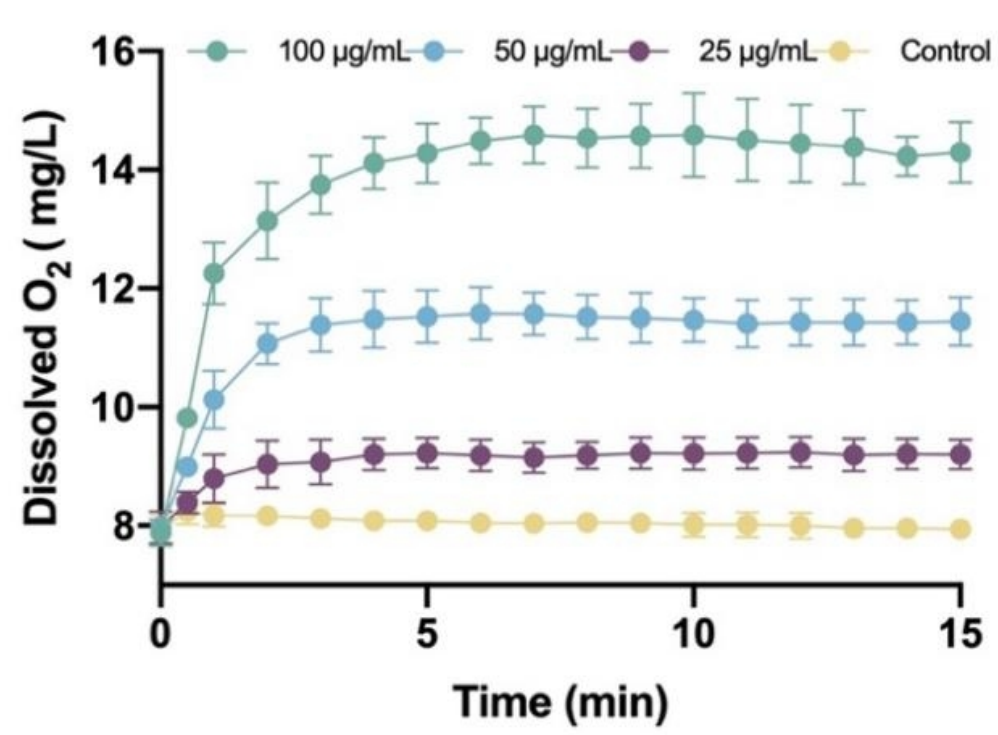

Fig. S2. O_2 release behavior of mPDA-C@M. O_2 generation of mPDA-C@M with different concentrations in 100 mM H_2O_2 solution.

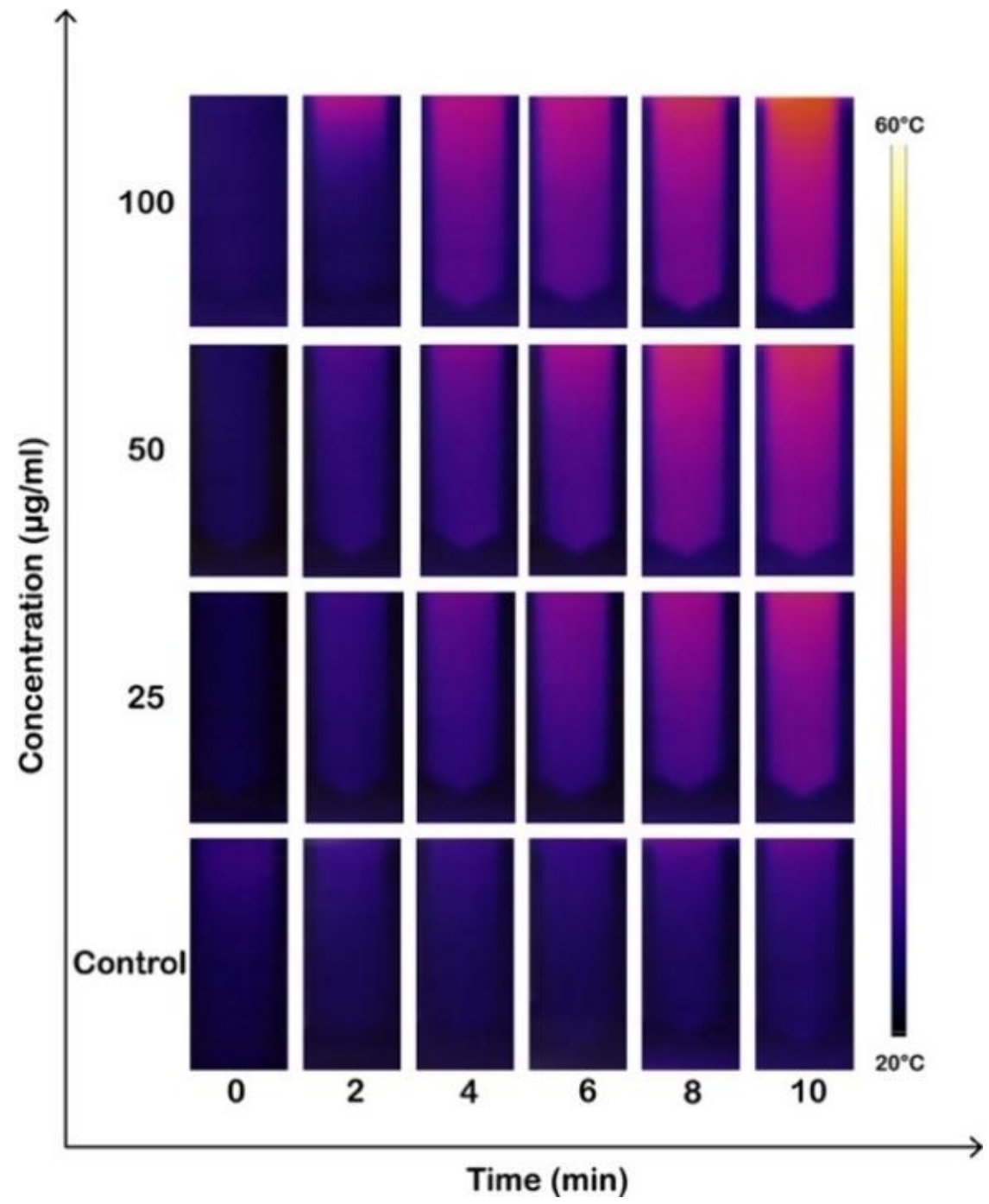

Fig. S3. Infrared thermal images of mPDA-C@M. Infrared thermal images of mPDA-C@M (0-100 μg/mL) under 808 nm NIR irradiation (1 W/cm^2).

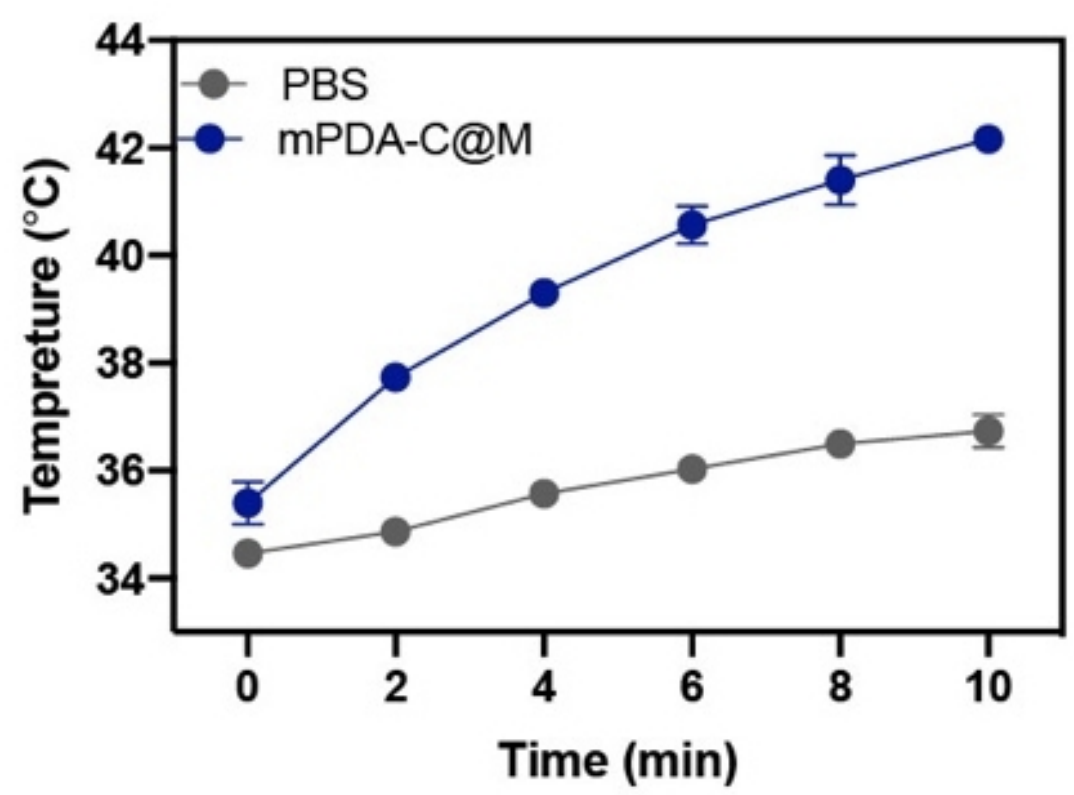

Fig. S4. Temperature variations over time in knee joints. Comparative in vivo thermal curves with mPDA-C@M administration under NIR irradiation (n = 3, mean ± SD).

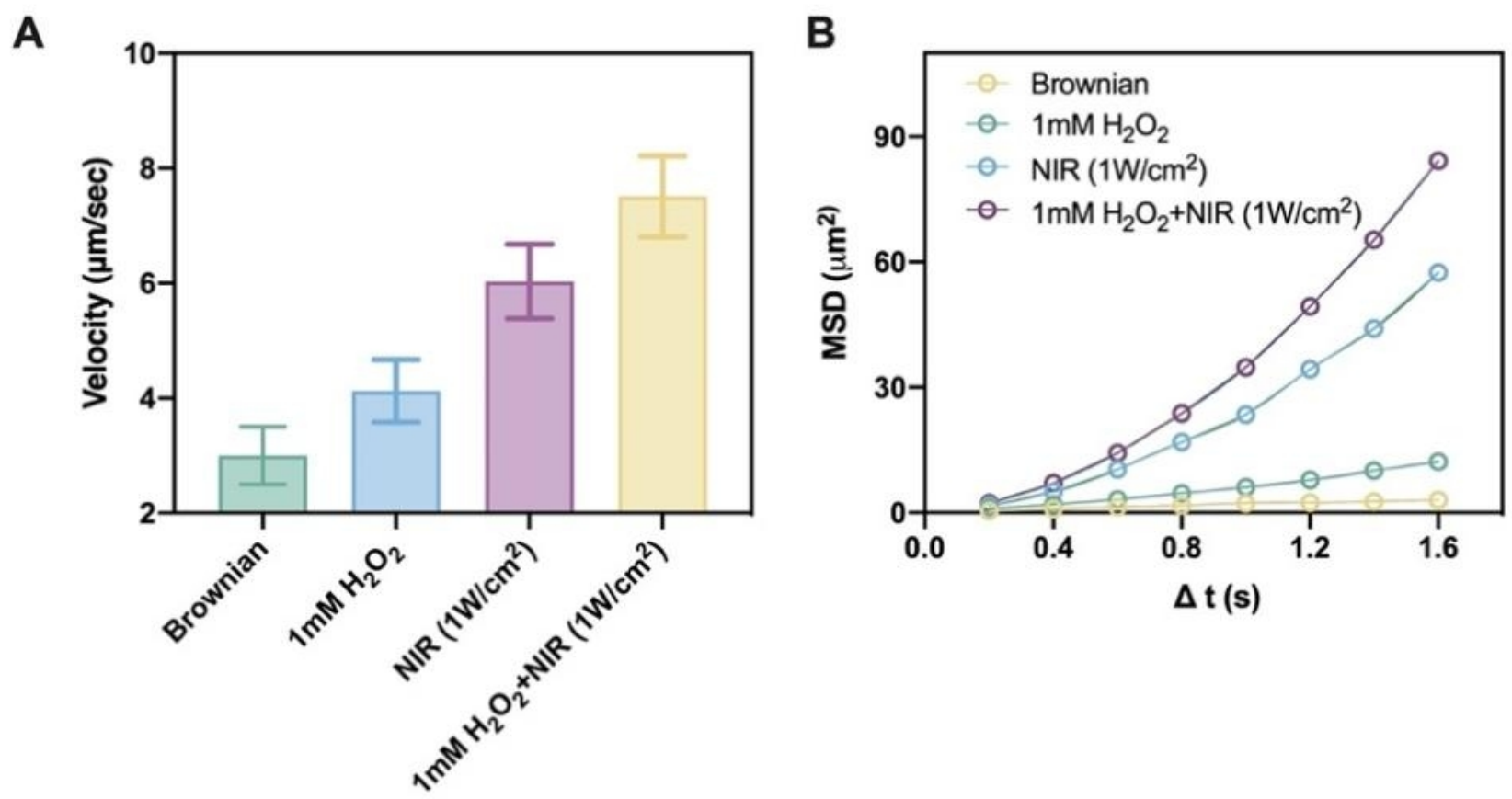

Fig S5. Quantitative comparison of motility under different conditions. (A) Average velocity of mPDA-C@M under different propulsion modes. (B) MSD curves of mPDA-C@M under different propulsion modes.

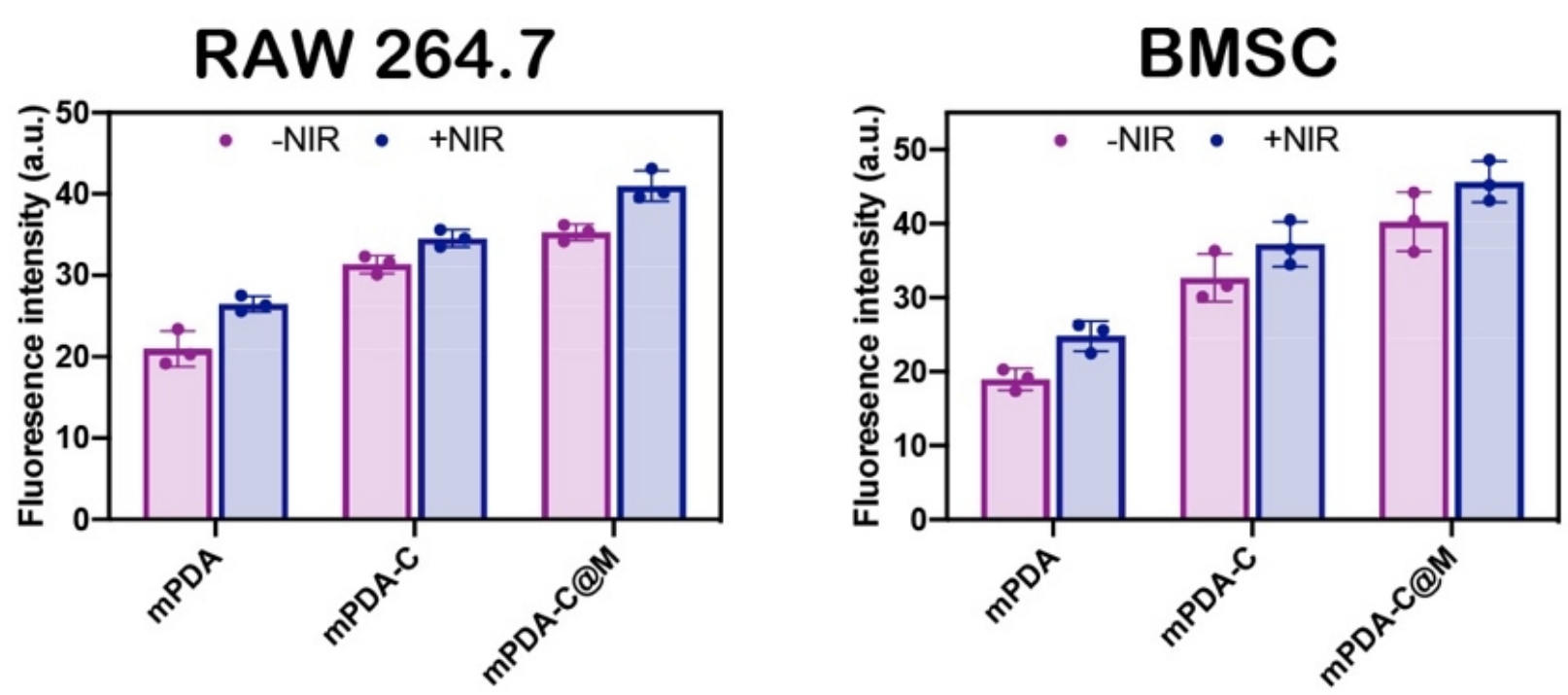

Fig. S6. Intracellular uptake analysis of mPDA-C@M. Corresponding fluorescence intensity analysis in RAW 264.7 cells and BMSCs.

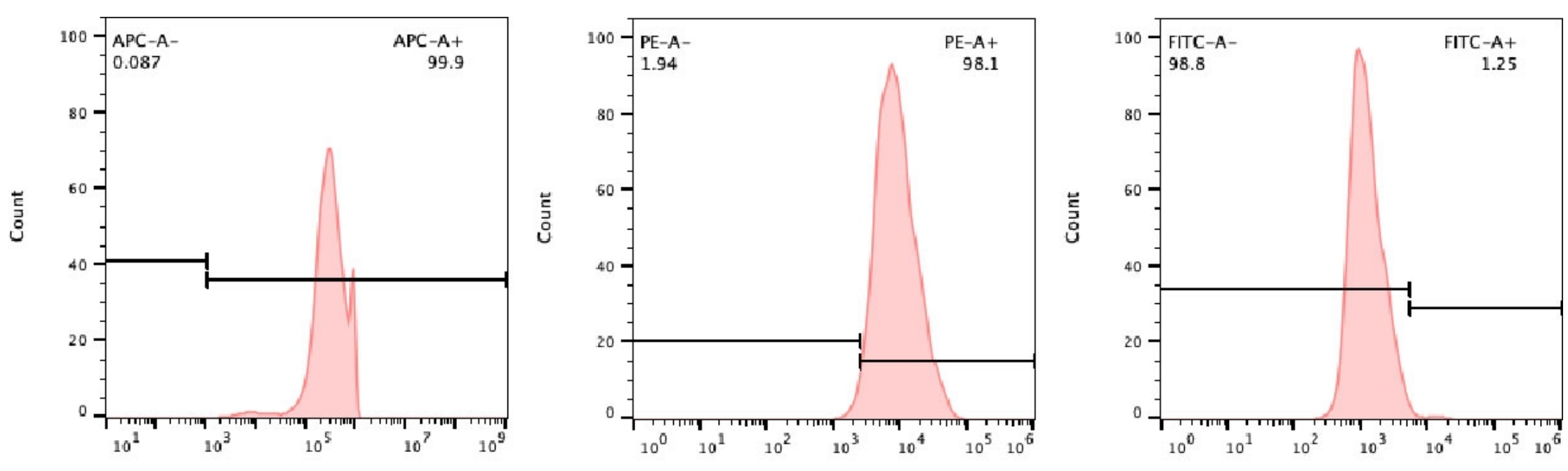

Fig. S7. Flow cytometry analysis of BMSCs. Flow cytometry analysis of BMSCs for the expression of positive surface markers (CD90, CD44) and negative marker (CD45).

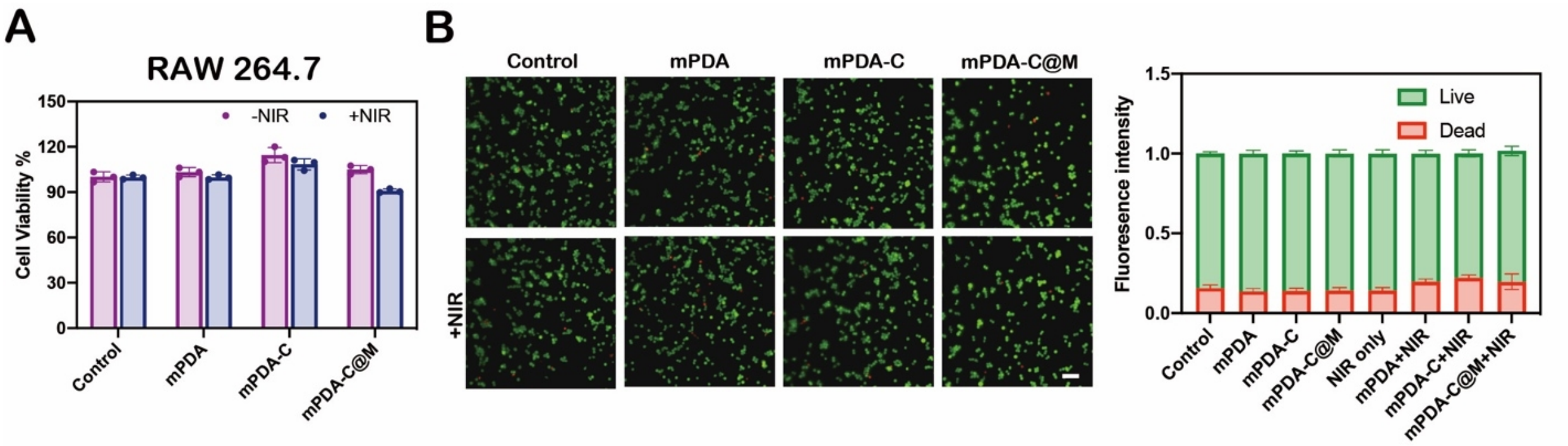

Fig. S8. Cell Viability Assessment of RAW 264.7 Macrophages. (A) Cell proliferation of RAW 264.7 macrophages detected by the CCK-8 assay. (B) Live/Dead staining images and corresponding fluorescence intensity analysis of RAW 264.7 macrophages following 24 h of co-culture with mPDA-C@M (n = 3, mean ± SD). Scale bar: 50 μm.

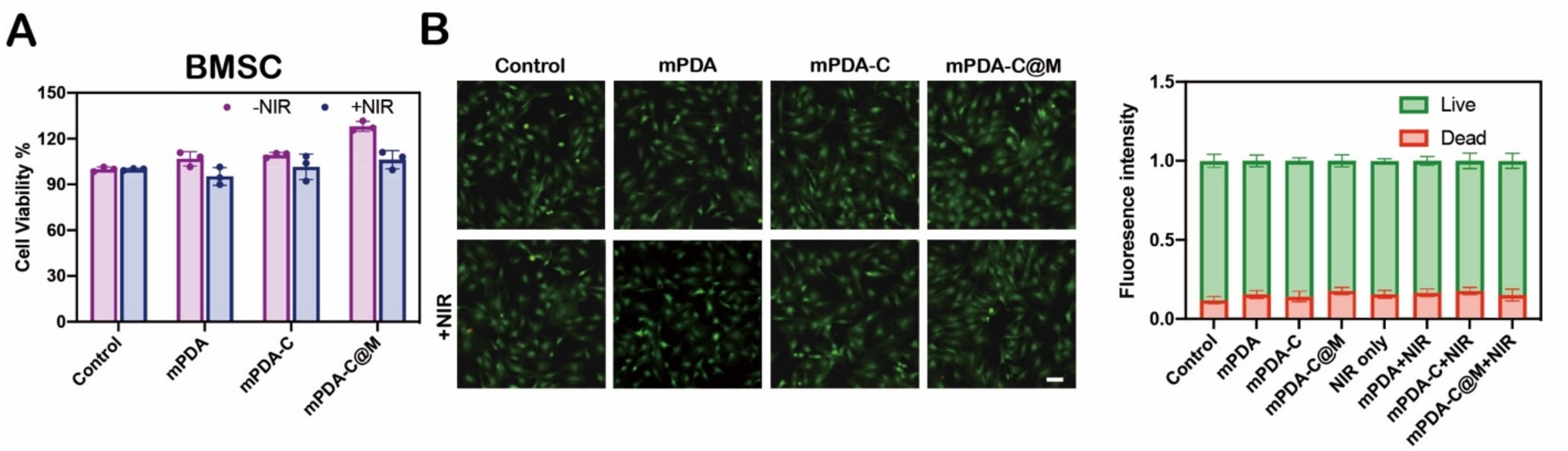

Fig. S9. Cell Viability Assessment of BMSCs. (A) Cell proliferation of RAW 264.7 macrophages detected by the CCK-8 assay. (B) Live/Dead staining images and corresponding fluorescence intensity analysis of BMSCs following 24 h of co-culture with mPDA-C@M (n = 3, mean ± SD). Scale bar: 50 μm.

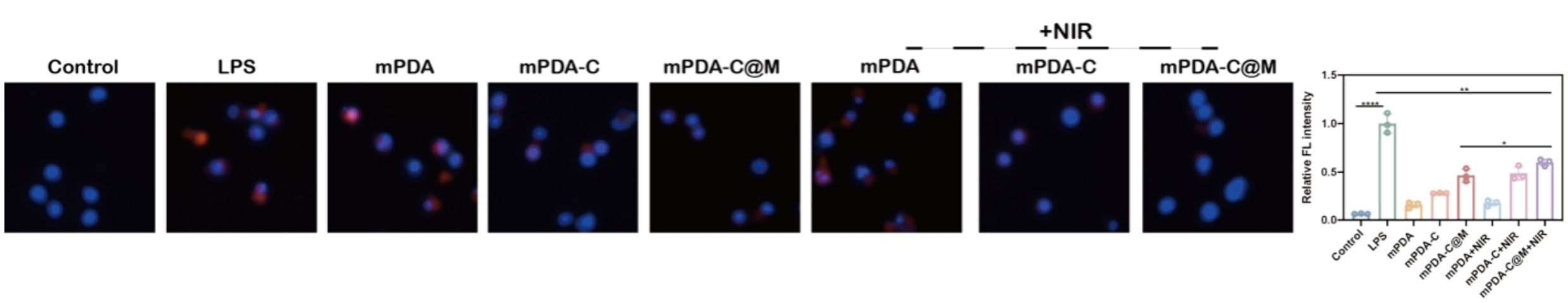

Fig. S10. Intracellular O_2 generation in RAW264.7 macrophages. $Ru(dpp)_3Cl_2$ probe staining images and corresponding fluorescence intensity analysis following of co-culture with mPDA-C@M (n = 3, mean ± SD). The red fluorescence of the dye was quenched in the presence of O_2. Scale bar: 50 μm.

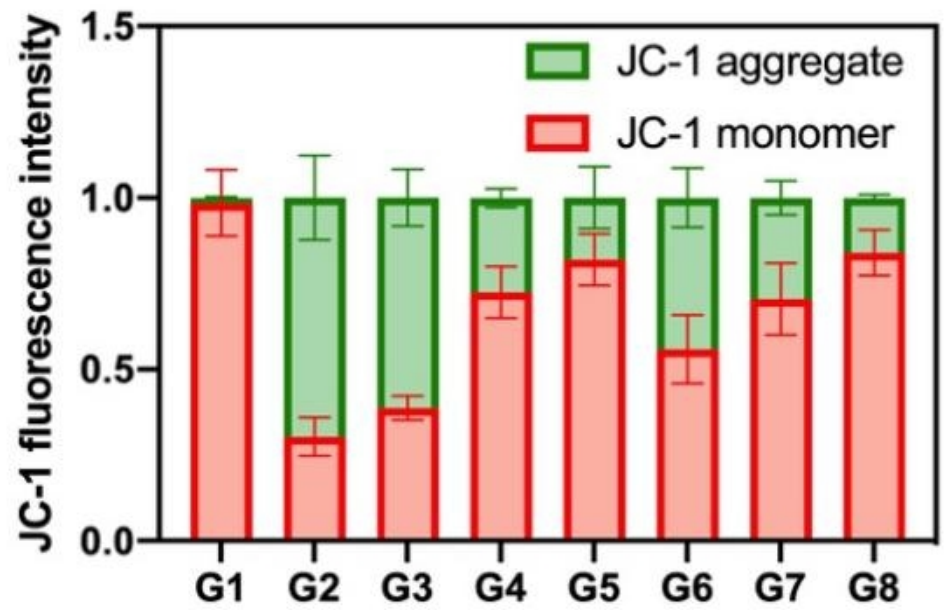

Fig. S11. Fluorescence intensity analysis of JC-1 staining. Fluorescence intensity analysis following of co-culture with mPDA-C@M and quantitative fluorescence intensity (n = 3, mean ± SD).

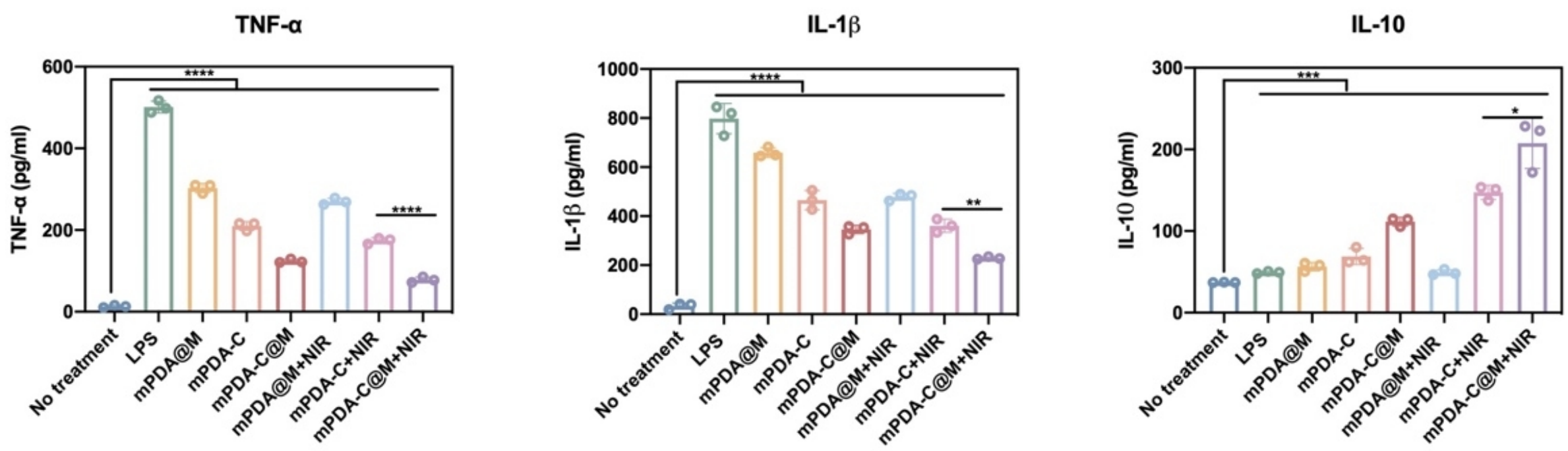

Fig. S12. Modulation of macrophage inflammatory cytokine secretion. ELISA quantification of TNF-α, IL-1β and IL-10 in the supernatants of LPS-stimulated RAW264.7 cells after various treatments (n = 3, mean ± SD). *P < 0.05, **P < 0.01, ***P < 0.001, and ****P < 0.0001.

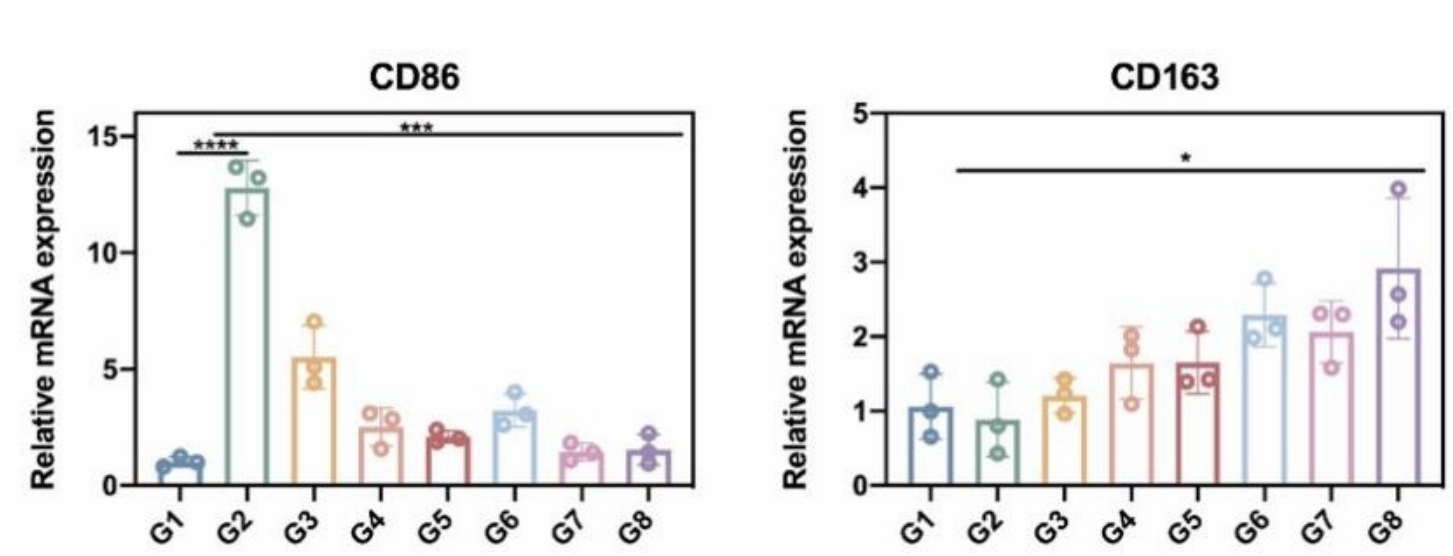

Fig. S13. Transcriptional analysis of BMSCs. Relative mRNA expression levels of CD86 and CD163in LPS-induced RAW 264.7 cells (n = 3, mean ± SD). *P < 0.05, **P < 0.01, ***P < 0.001, and ****P < 0.0001.

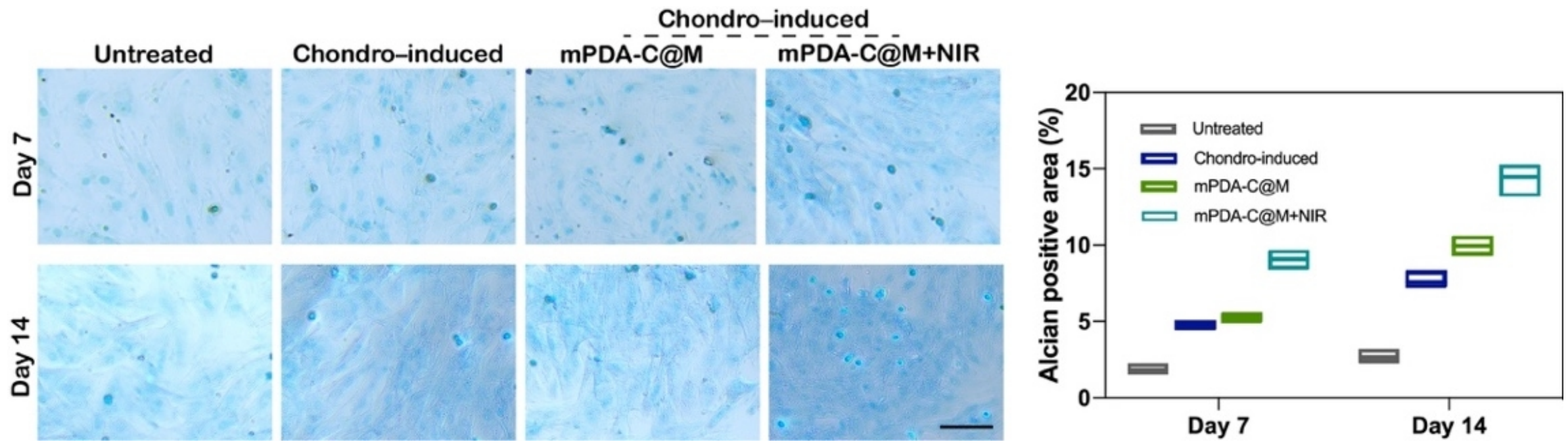

Fig. S14. Alcian blue staining of BMSCs. Alcian blue staining of BMSCs with different treatment for 7 days and 14 days and corresponding fluorescence intensity analysis (n = 3, mean ± SD). Scale bar = 100 μm.

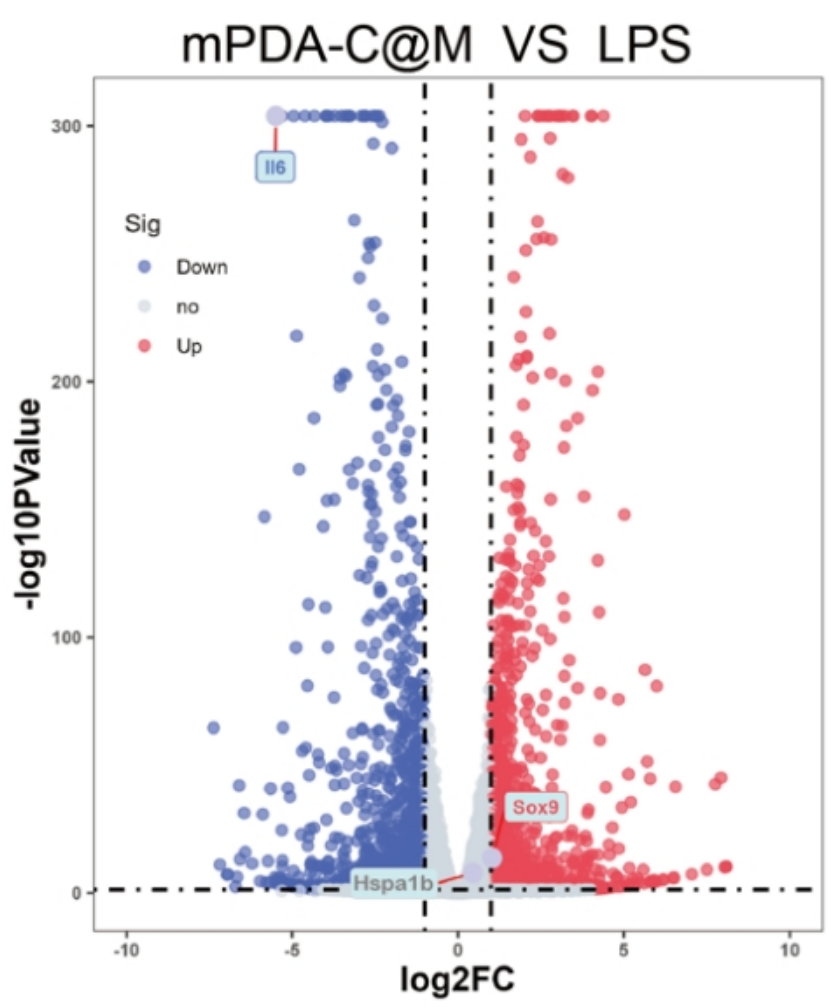

Fig. S15. Identification of Differentially Expressed Genes. Volcano plots of differentially expressed genes (DEGs) showing comparisons between mPDA-C@M-treated BMSCs vs. LPS group (fold change ⩾ 1 and P ⩽ 0.05).

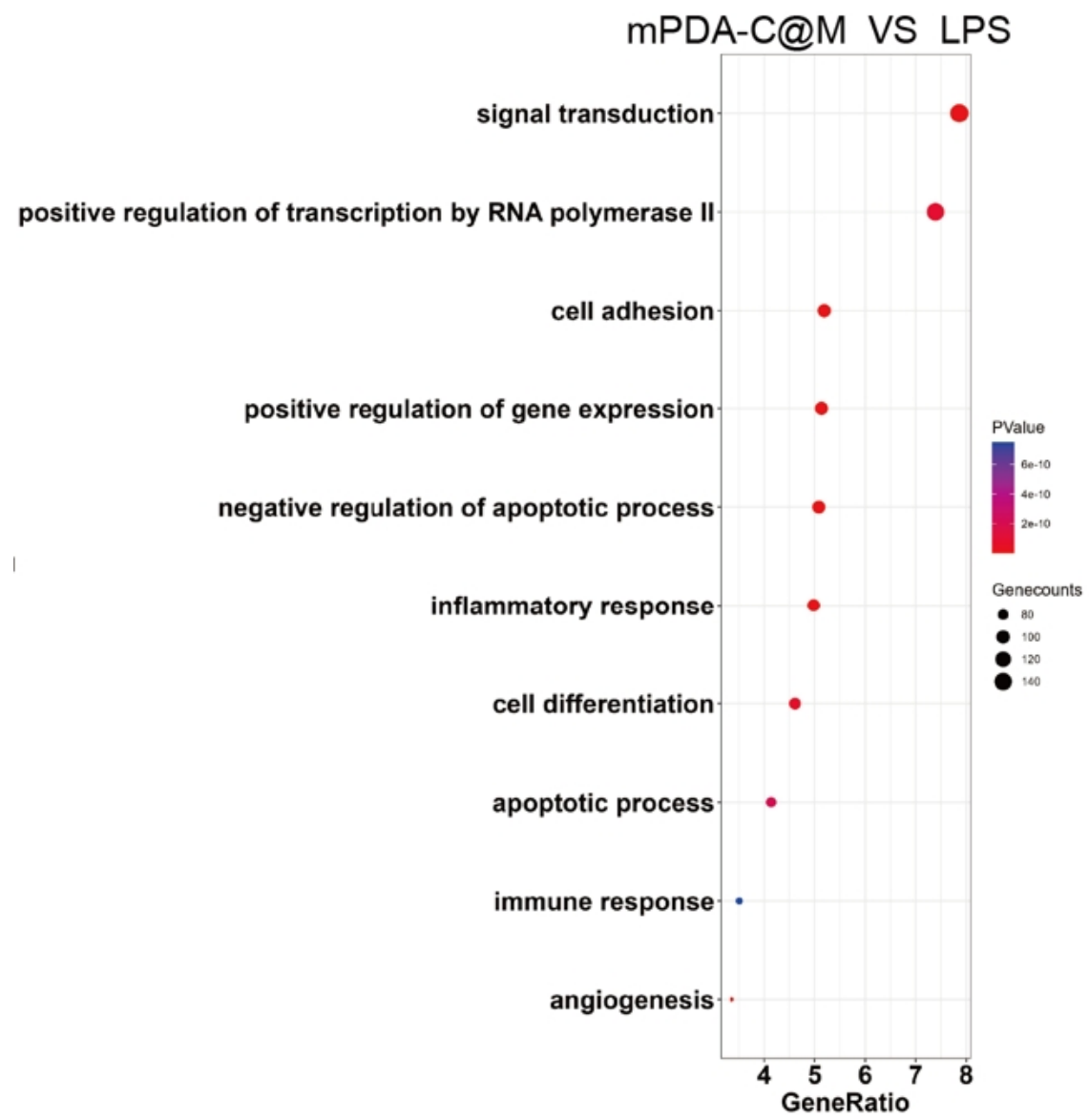

Fig. S16. GO Enrichment Analysis of Transcriptomic Changes (mPDA-C@M vs. LPS). The GO analysis between mPDA-C@M-treated BMSCs and LPS-treated BMSCs.

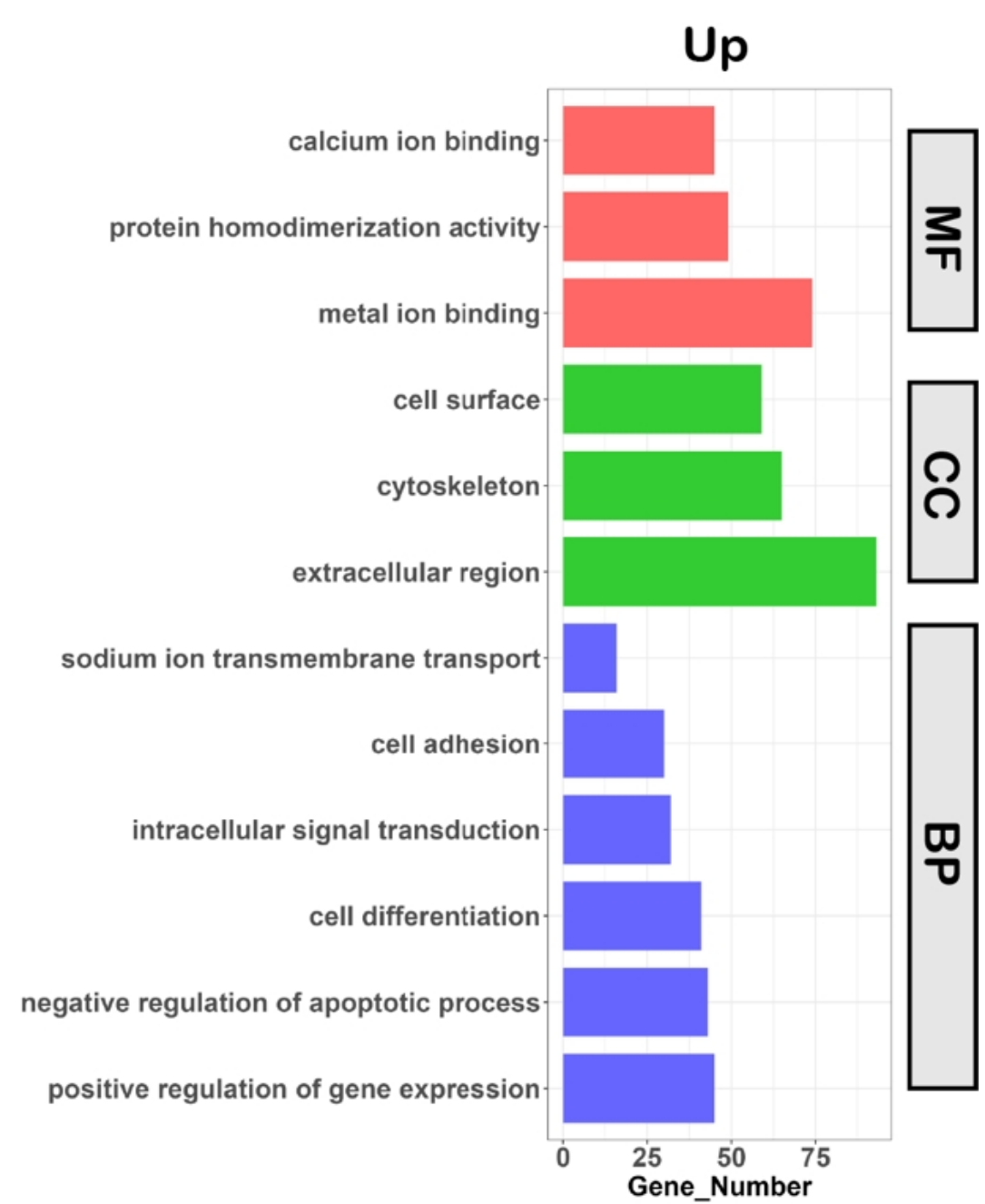

Fig. S17. GO Enrichment Analysis of Transcriptomic Changes (mPDA-C@M+NIR vs. LPS). The GO analysis between mPDA-C@M+NIR-treated BMSCs and LPS-treated BMSCs.

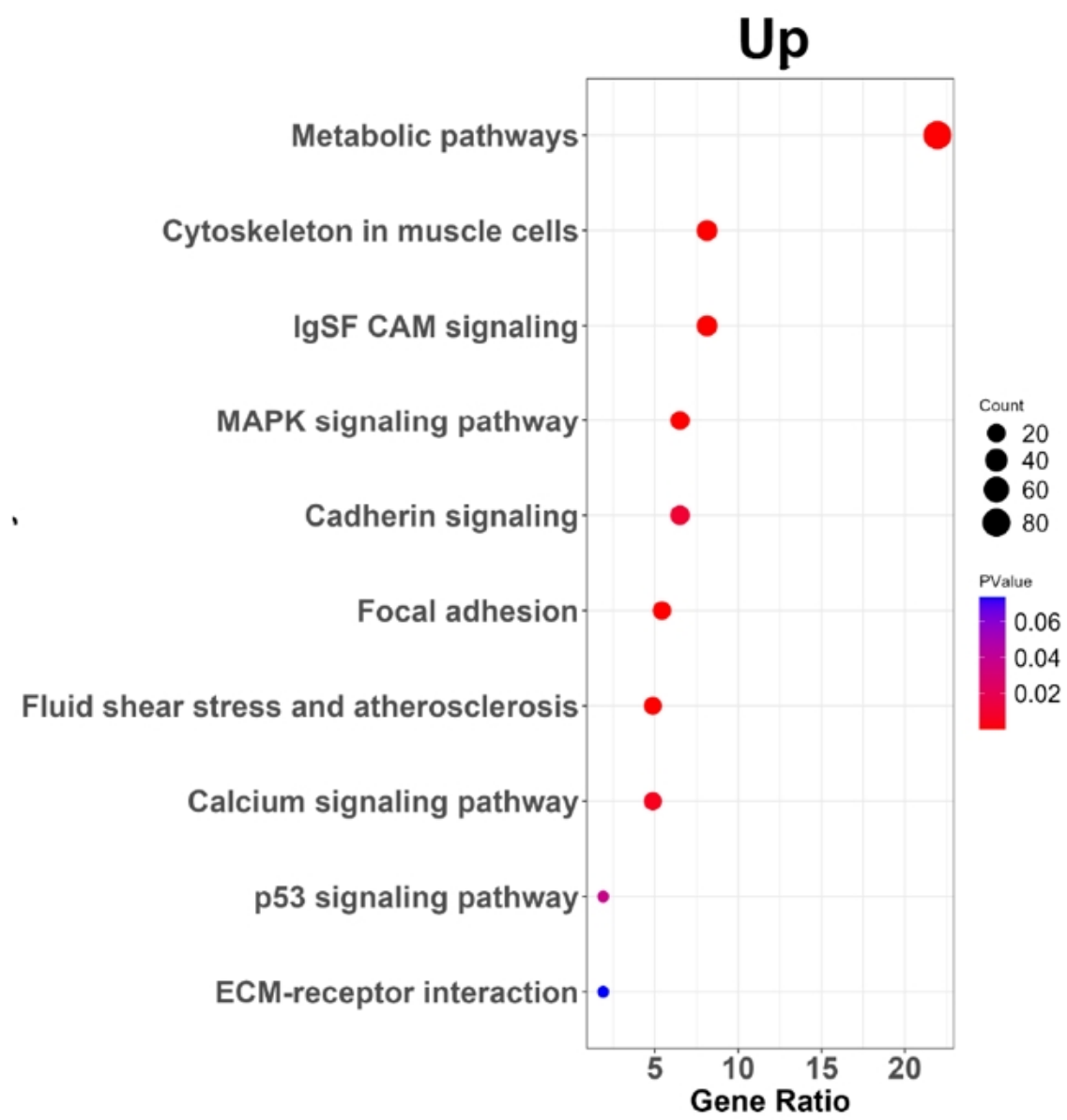

Fig. S18. KEGG Pathway Enrichment Analysis. The KEGG enrichment analysis of upregulated pathways between mPDA-C@M+NIR-treated BMSCs and LPS-treated BMSCs.

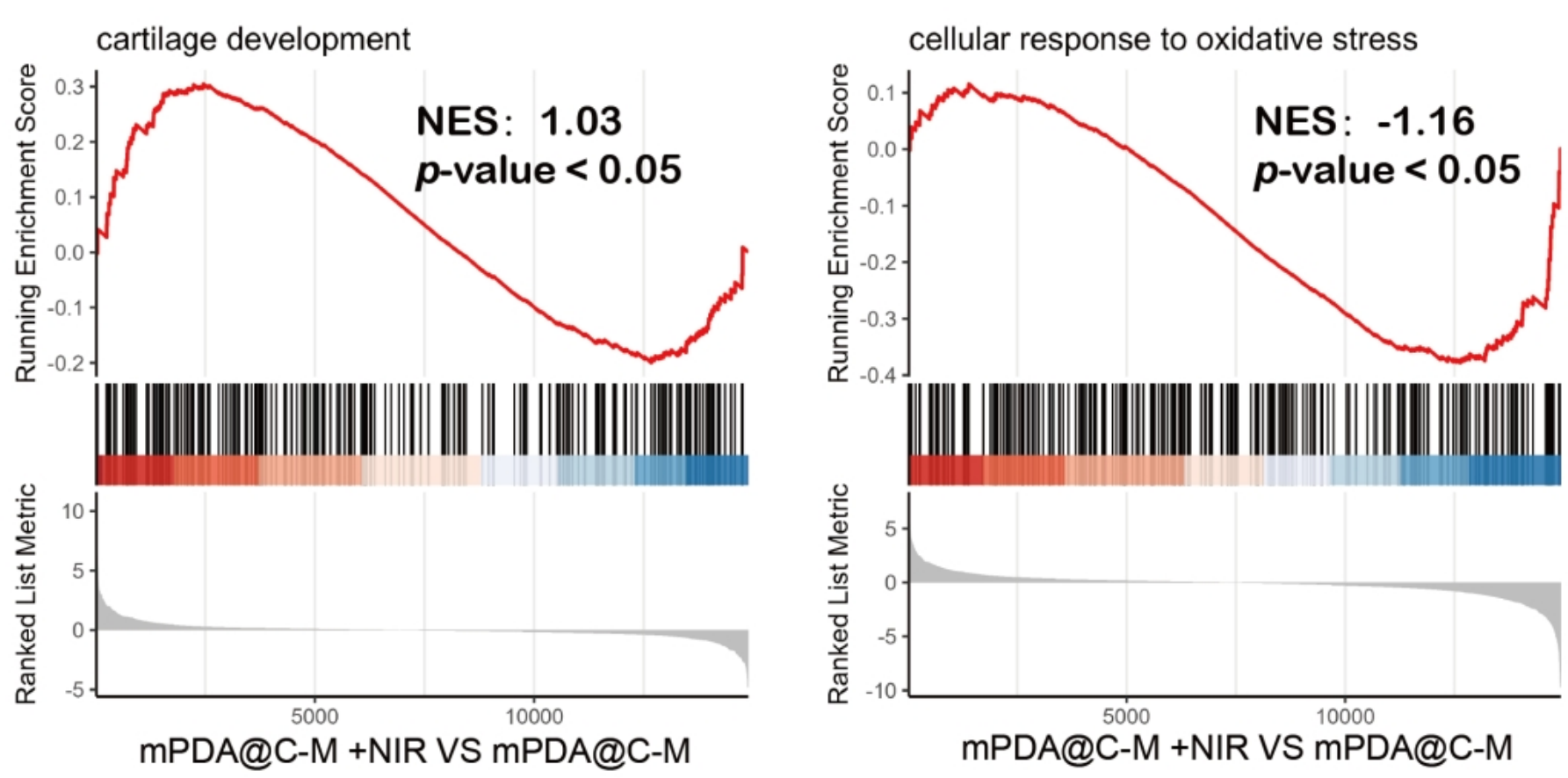

Fig. S19. The GSEA between mPDA-C@M+NIR-treated BMSCs and mPDA-C@M-treated BMSCs. The GSEA to compare the gene sets related to (A)cartilage development and (B) cellular response to oxidative stress between mPDA-C@M+NIR-treated BMSCs and mPDA-C@M-treated BMSCs.

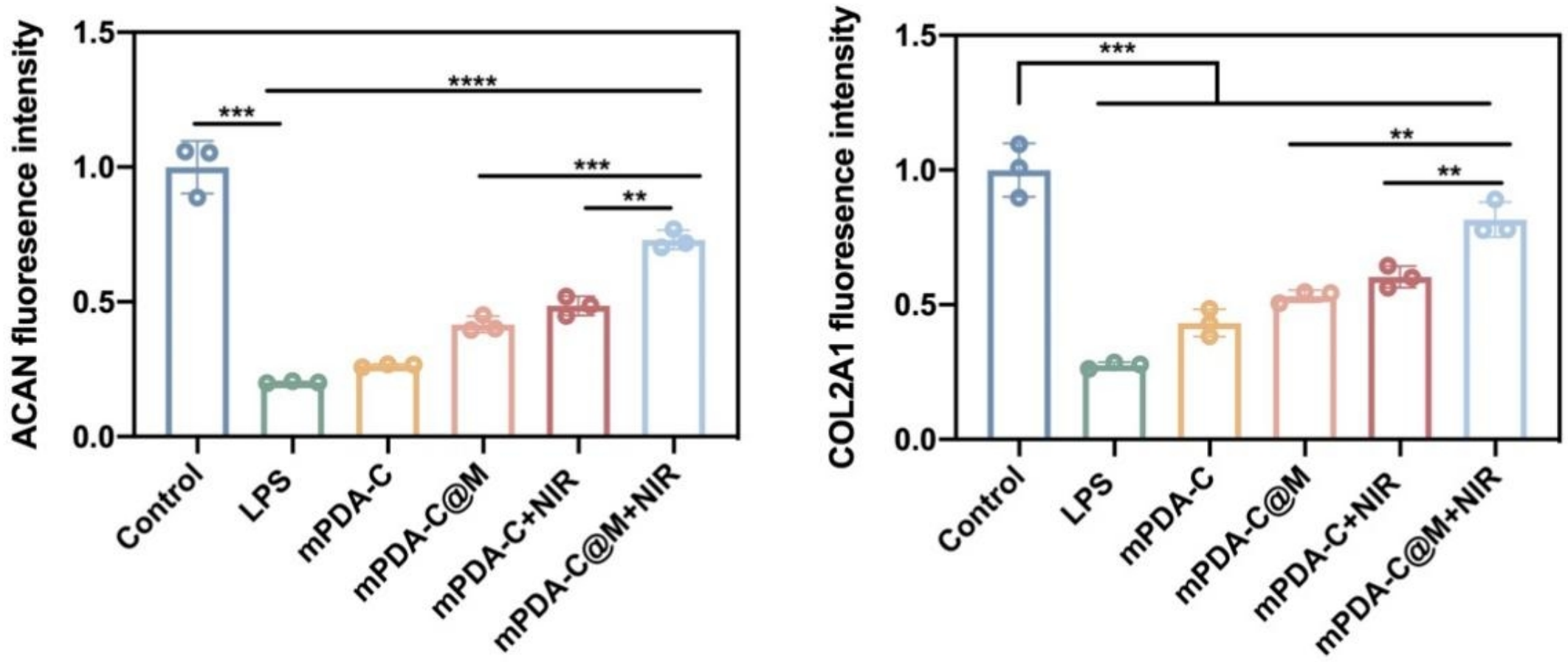

Fig. S20. Immunofluorescent staining analysis of BMSCs. Corresponding fluorescence intensity analysis of ACAN and COL2A1 in BMSCs (n = 3, mean ± SD). *P < 0.05, **P < 0.01, ***P < 0.001, and ****P < 0.0001.

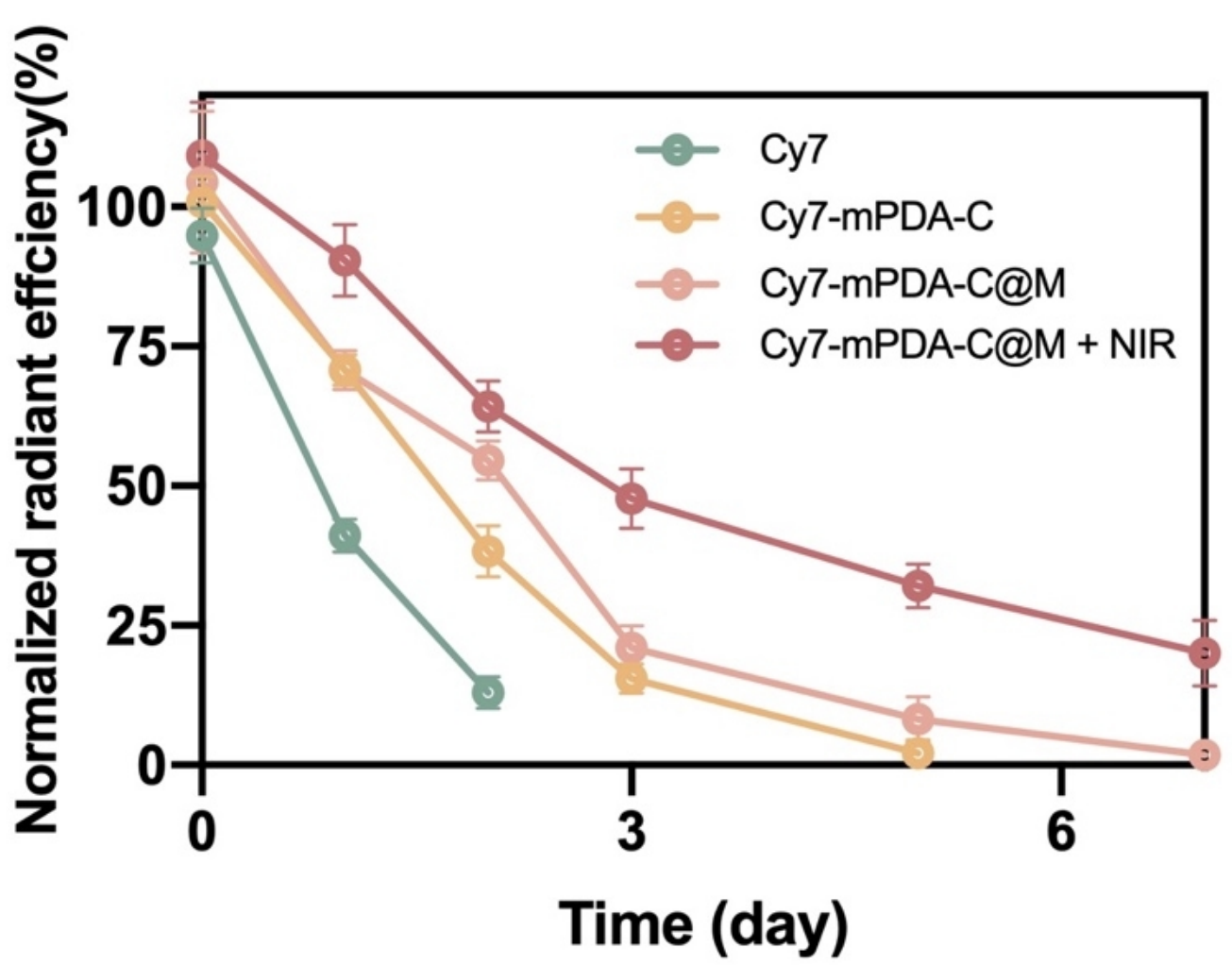

Fig. S21. Quantitative analysis of in vivo fluorescence retention. Time-dependent decay of fluorescence intensity in the knee joints of MIA-induced OA rats following a single intra-articular injection of different Cy 7-labled formulations. Data correspond to the images in Fig. 6A (n = 3, mean ± SD).

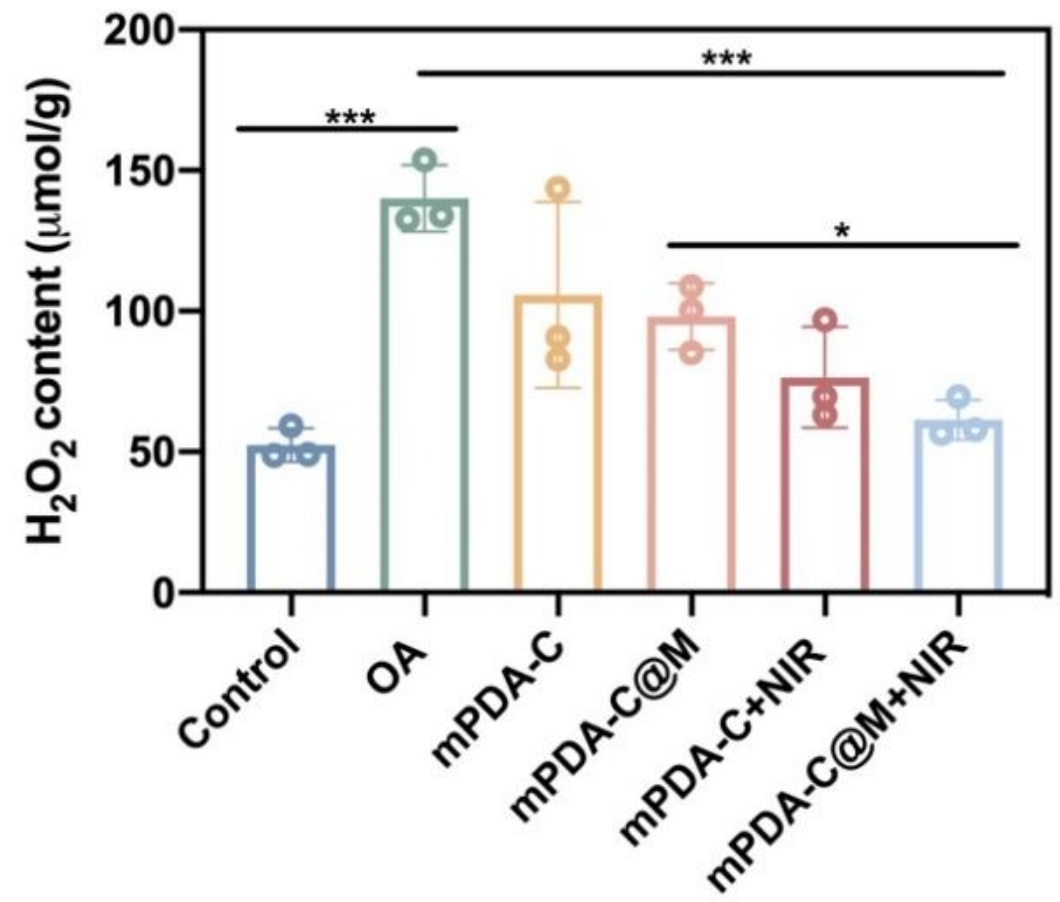

Fig. S22. **H_2O_2 content in OA joint cavity.** H_2O_2 level in the synovium tissue of MIA-induced OA rats following IA injection of different formulations (n = 3, mean ± SD). *P < 0.05, **P < 0.01, ***P < 0.001, and ****P < 0.0001.

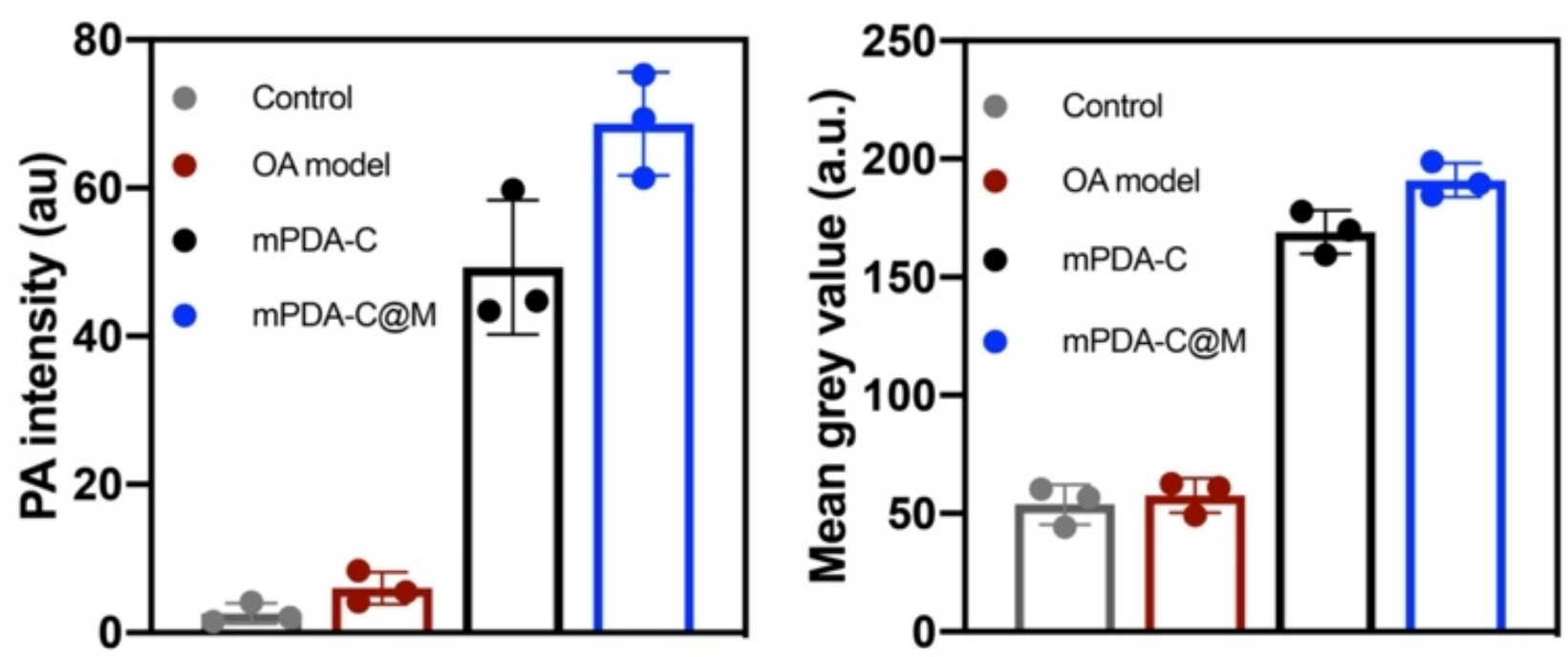

Fig. S23. Intensity quantification of PA/US images. Signal intensity quantification of dual-modal PA/US images of the joint in MIA-induced rats (n = 3, mean ± SD).

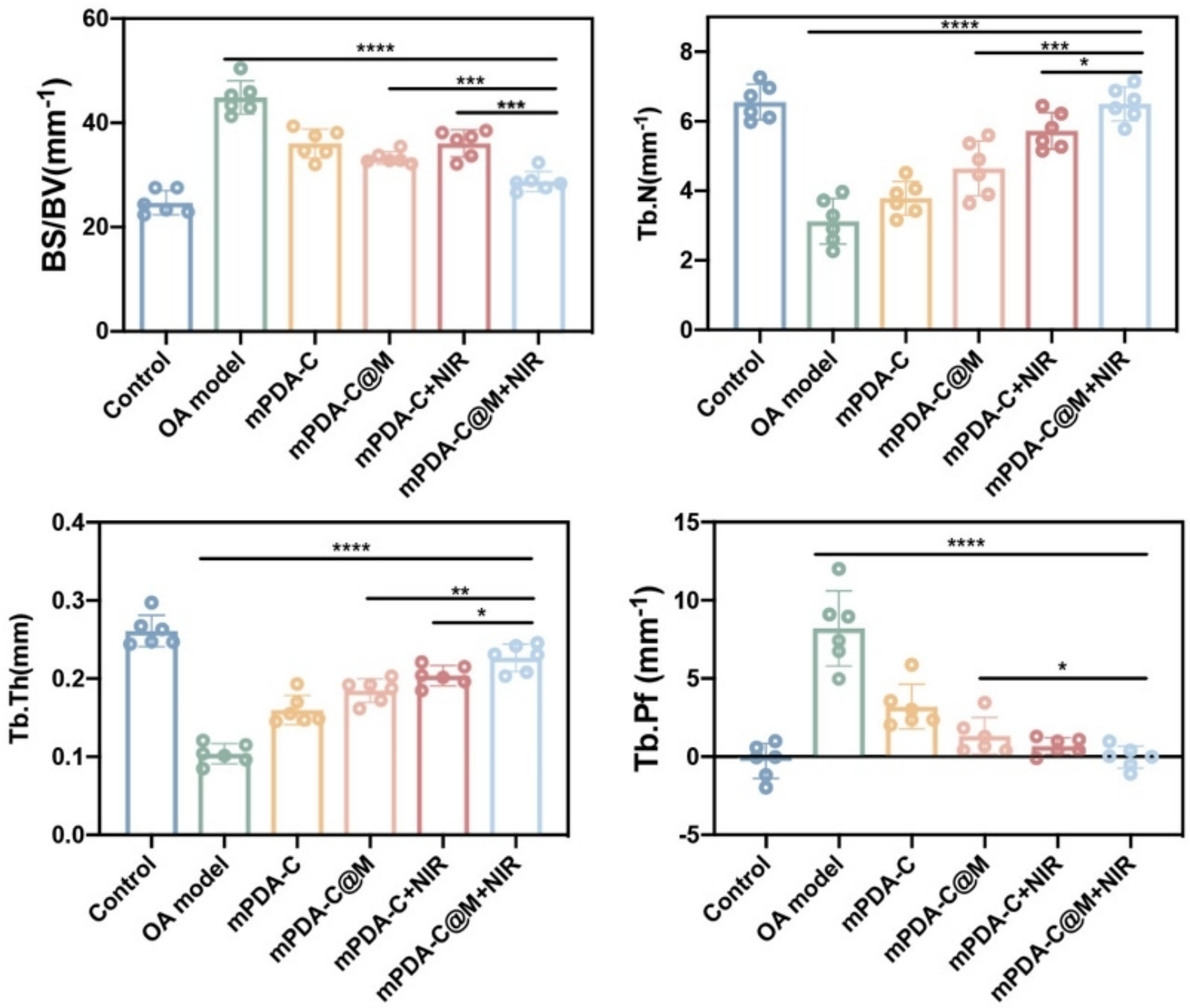

Fig. S24. mPDA-C@M+NIR alleviate joint degradation and damage in MIA-induced rats. Related parameters (bone surface-to-volume ratio (BS/BV), trabecular thickness (Tb. Th), trabecular number (Tb. N), and trabecular pattern factor (Tb. Pf)) of tibial subchondral bone microarchitecture were quantified (n = 6, mean ± SD). *P < 0.05, **P < 0.01, ***P < 0.001, and ****P < 0.0001.

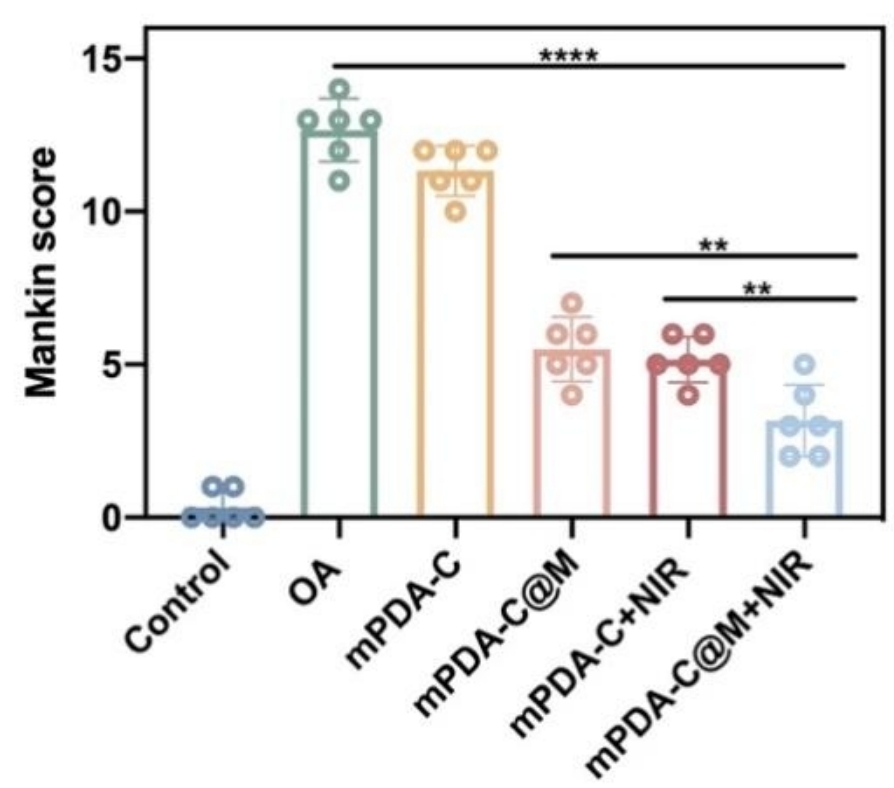

Fig. S25. mPDA-C@M+NIR alleviate joint degradation and damage in MIA-induced rats. Mankin score was assessed for each treatment (n = 6, mean ± SD). *P < 0.05, **P < 0.01, ***P < 0.001, and ****P < 0.0001.

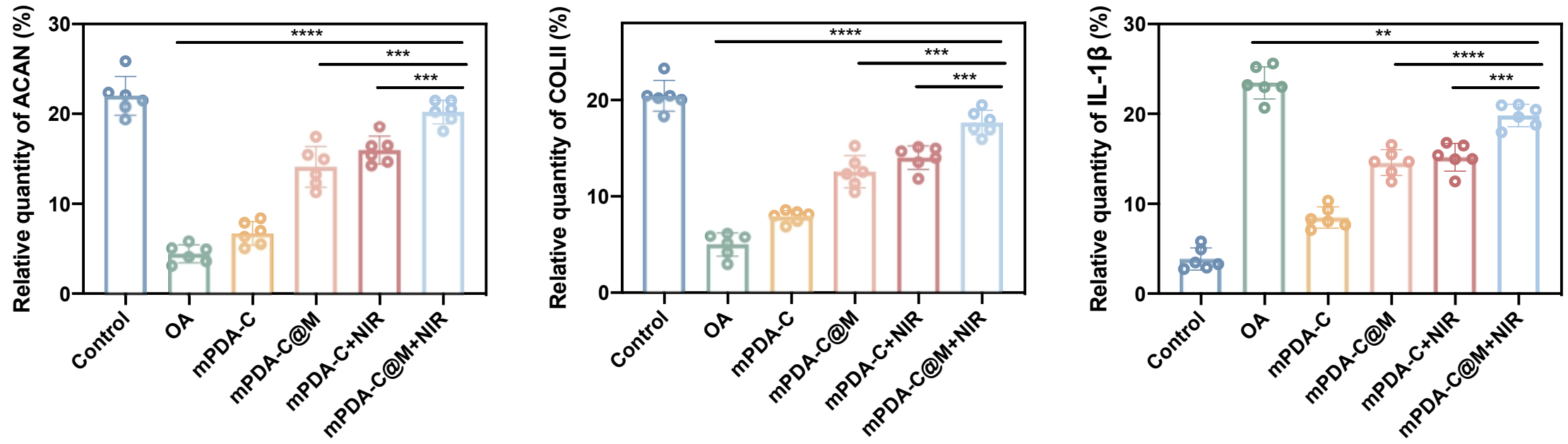

Fig. S26. mPDA-C@M+NIR alleviate joint degradation and damage in MIA-induced rats. Semi-quantification of the protein levels of ACAN, COLII and IL-1β in the cartilage from different groups (n = 6, mean ± SD). *P < 0.05, **P < 0.01, ***P < 0.001, and ****P < 0.0001.

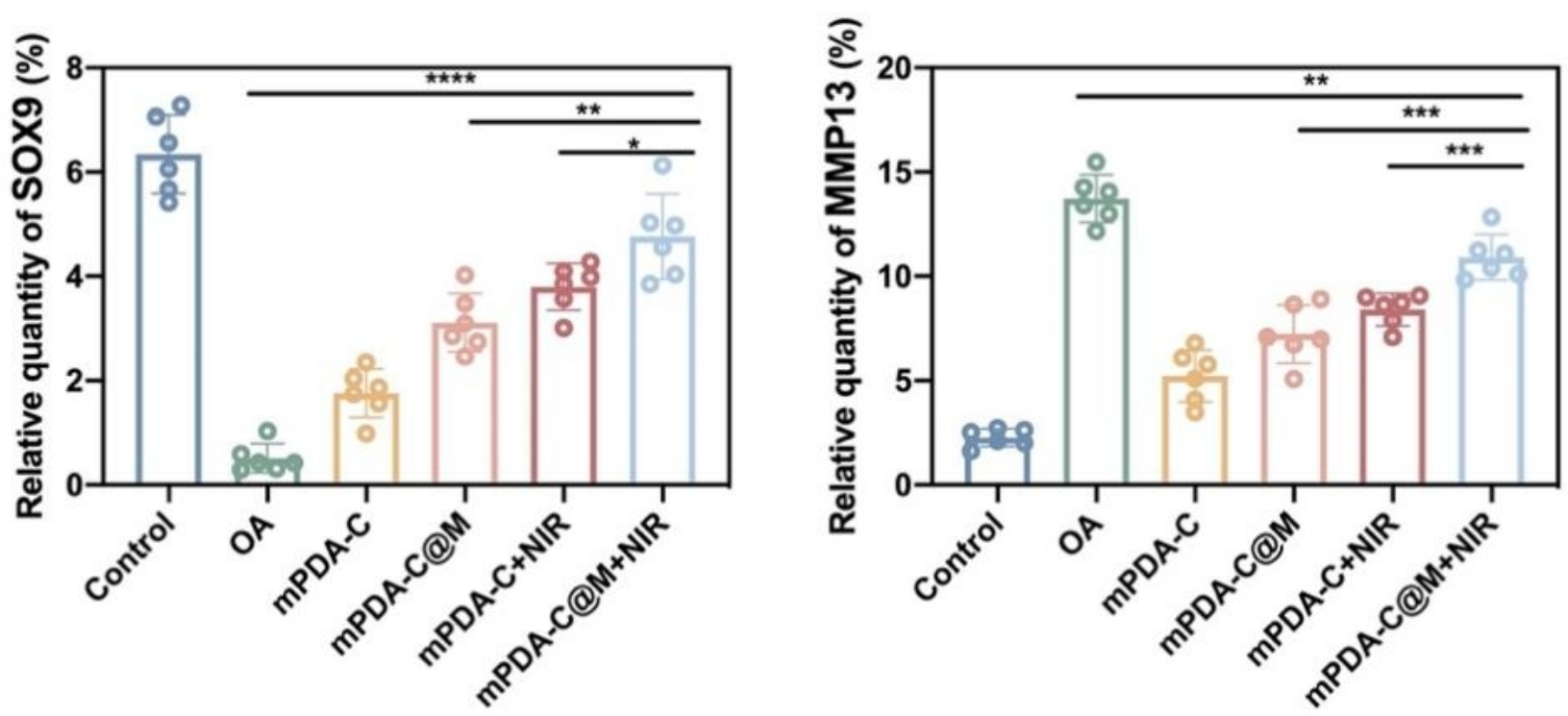

Fig. S27. mPDA-C@M+NIR alleviate joint degradation and damage in MIA-induced rats. Semi-quantification of the protein levels of SOX9 and MMP13 in the cartilage from different groups (n = 6, mean ± SD). *P < 0.05, **P < 0.01, ***P < 0.001, and ****P < 0.0001.

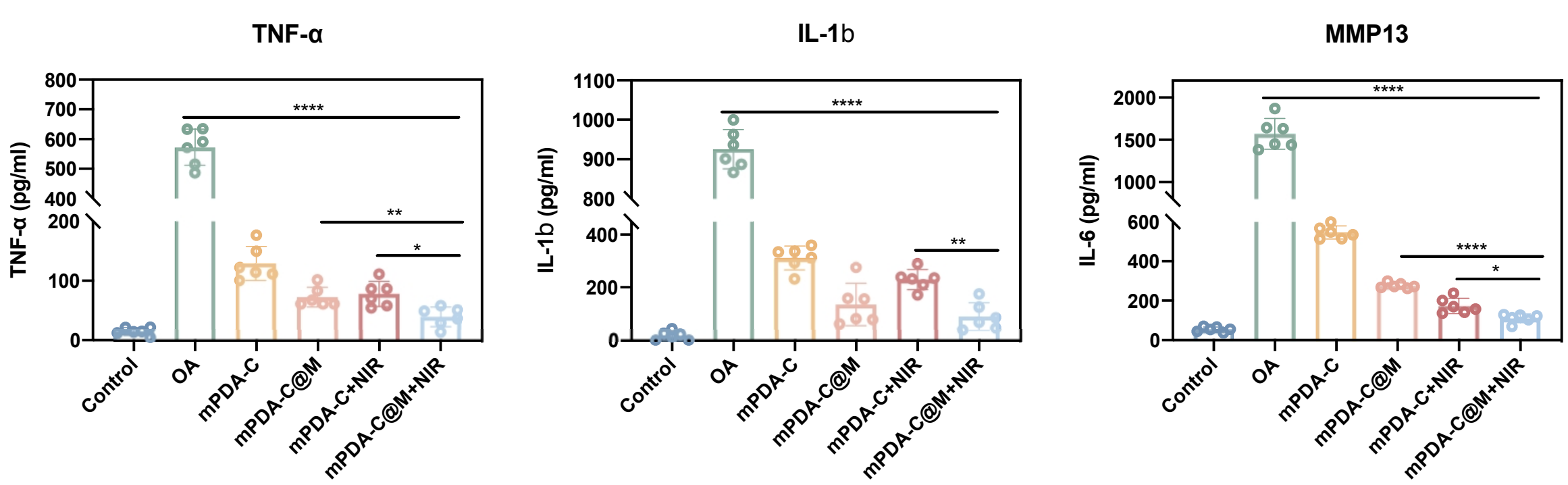

Fig. S28. mPDA-C@M+NIR alleviate joint inflammation in MIA-induced rats. Quantitative analysis of serum levels (TNF-α, IL-1β and MMP13) from different groups (n = 6, mean ± SD). *P < 0.05, **P < 0.01, ***P < 0.001, and ****P < 0.0001.

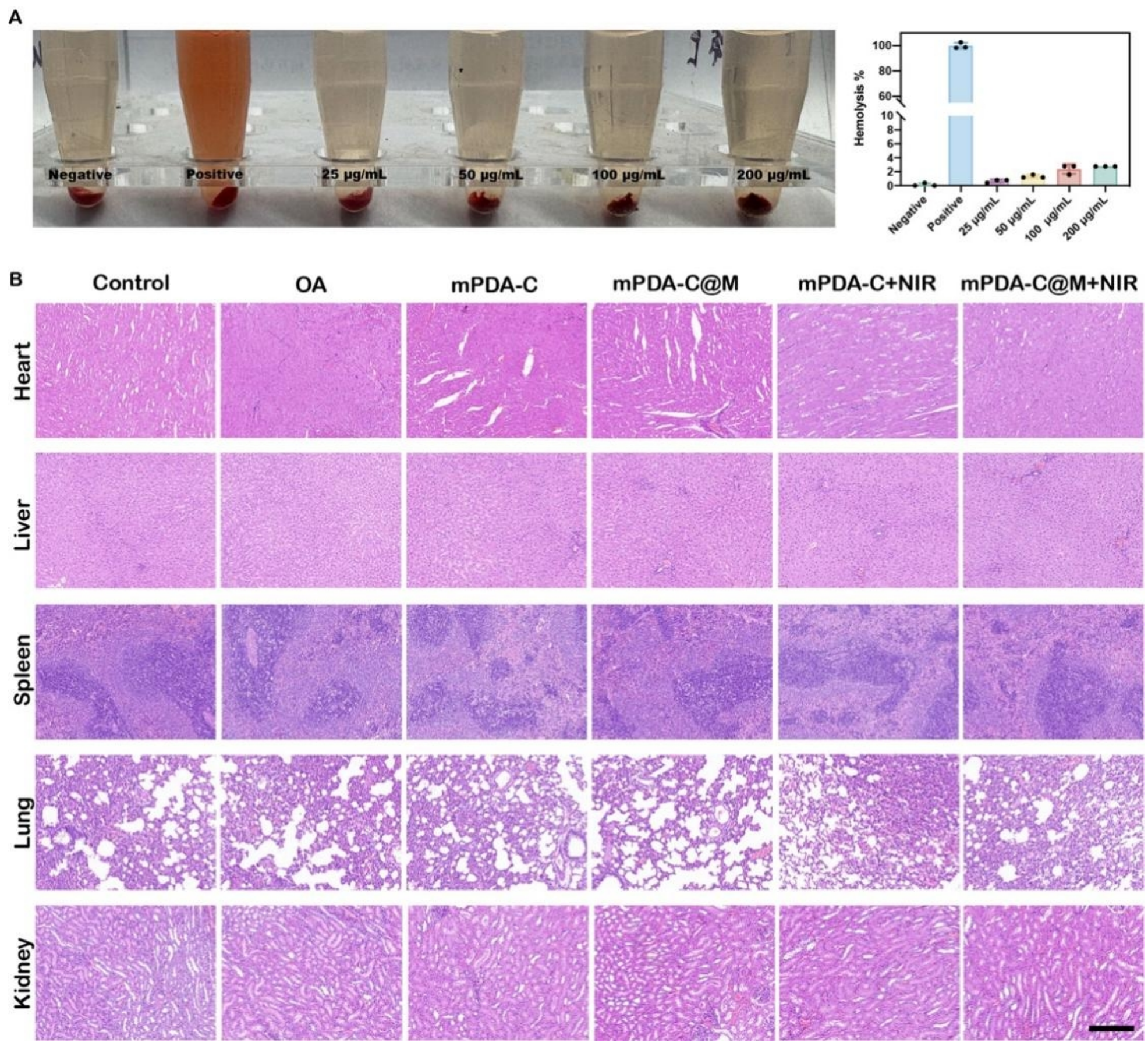

Fig. S29. Evaluation of systemic toxicity of mPDA-C@M MIA-induced rats. (A) Photographs and hemolysis assay of negative, positive and mPDA-C@M with different concentrations. (B) Biosafety assessment of heart, liver, spleen, lung, and kidney indifferent groups measured by H&E staining. Scale bar, 100 µm.

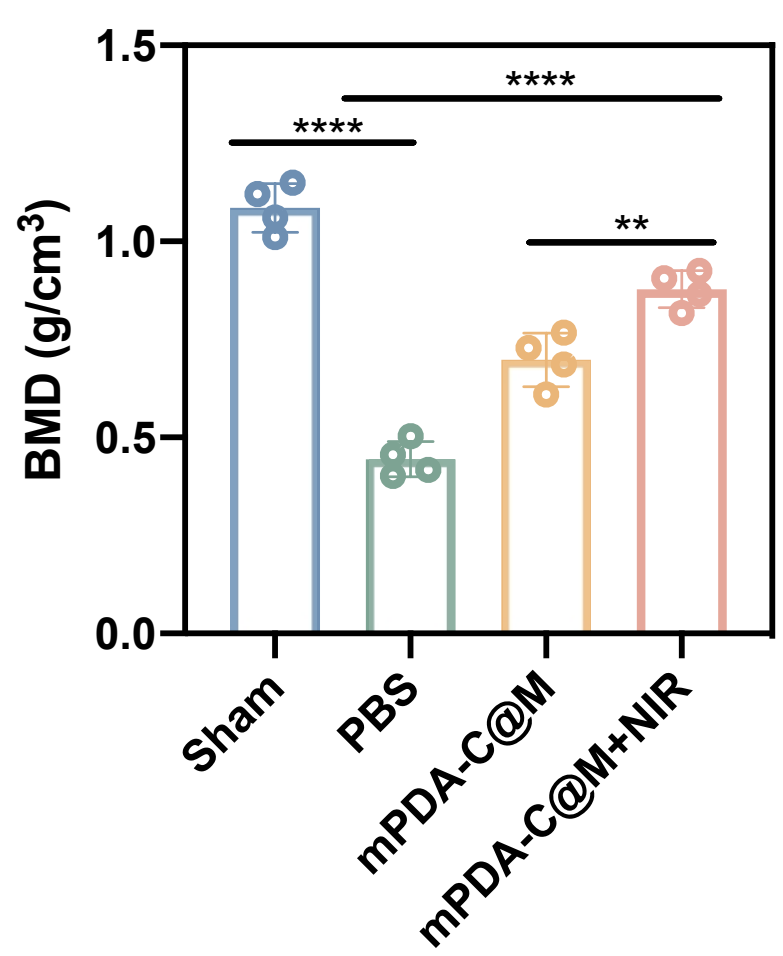

Fig. S30. mPDA-C@M+NIR enhance cartilage regeneration in rabbit cartilage defect model. BMD per joint were quantified (n = 4, mean ± SD). *P < 0.05, **P < 0.01, ***P < 0.001, and ****P < 0.0001.

Supplementary Tables

Table S1 Full description of genes in the cluster heatmap from Novogene platform.

gene	Full description
Hapln1	hyaluronan and proteoglycan link protein 1 [Source:RGD Symbol;Acc:2412]
Hspa1b	heat shock protein family A (Hsp70) member 1B [Source:RGD Symbol;Acc:2840]
Hspa1a	heat shock protein family A (Hsp70) member 1A [Source:RGD Symbol;Acc:2839]
Cspg4	chondroitin sulfate proteoglycan 4 [Source:RGD Symbol;Acc:619942]
Sox9	SRY box 9 [Source:RGD Symbol;Acc:620474]
Acan	aggrecan [Source:RGD Symbol;Acc:68358]
Hspb7	heat shock protein family B (small) member 7 [Source:RGD Symbol;Acc:62021]
Col2a1	collagen type II alpha 1 chain [Source:RGD Symbol;Acc:2375]
Hspa4l	heat shock protein family A (Hsp70) member 4 like [Source:RGD Symbol;Acc:1306528]
Has2	hyaluronan synthase 2 [Source:RGD Symbol;Acc:2781]
Mmp13	matrix metallopeptidase 13 [Source:RGD Symbol;Acc:620196]
Il1b	interleukin 1 beta [Source:RGD Symbol;Acc:2891]
Tnf	tumor necrosis factor [Source:RGD Symbol;Acc:3125]
Il6	interleukin 6 [Source:RGD Symbol;Acc:2901]
Adamts5	ADAM metallopeptidase with thrombospondin type 1 motif, 5 [Source:RGD Symbol;Acc:735196]
Ptgs2	prostaglandin-endoperoxide synthase 2 [Source:RGD Symbol;Acc:3133]
Mmp3	matrix metallopeptidase 3 [Source:RGD Symbol;Acc:2910]
Mmp9	matrix metallopeptidase 9 [Source:RGD Symbol;Acc:621320]

Table S2 Primer sequences for RT–qPCR (RAW 264.7 macrophages)

Target gene	Forward primer sequence	Reverse primer sequence
**18S**	AACTTTCGATGGTAGTCGCCG	CCTTGGATGTGGTAGCCGTTT
**IL-10**	GAGAAGCATGGCCCAGAAATC	GAGAATCGATGACAGCGCC
**IL-6**	ATCCAGTTGCCTTCTTGGGACTGA	TAAGCCTCCGACTTGTGAAGTGGT
**TNF-α**	TCTCATGCACCACCATCAAGGACT	ACCACTCTCCCTTTGCAGAACTCA
**HIF-1α**	AAGTCTAGGGATGCAGCAC	CAAGATCACCAGCATCTAG
**CD86**	TCCTCCAGCAGTGGGAAACA	TTTGTAGGTTTCGGGTATCCT
**CD163**	CTCAGCGTCTCTGCTGTCAC	GGCCAGTCTCAGTTCCTTCT
**MMP13**	GCCATTACCAGTCTCCGAGG	TACGGTTGGGAAGTTCTGGC
**HSP70**	GGTGCTGACGAAGATAAACGAG	TCGATGGCCTCGAACAGAG

Table S3 Primer sequences for RT–qPCR (BMSCs)

Target gene	Forward primer sequence	Reverse primer sequence
**β-actin**	ACCCGCGAGTACAACCTTCTT	TCGTCATCCATGGCGAACTGG
**HSP70**	CGGTCTCCTAAAGGTCGACCAT	CGAACTTCTTGCGGCCCAGCT
**IL–1β**	CAACCAACAAGTGATATTCTCCATG	GATCCACATCTCCAGCTGCA
**MMP 13**	GGTGATGAAGATGATTTGTCTGAGG	CGTCAAGTTTGCCAGTCACCT
**TNF–α**	ATGAGCACAGAAAGCATGATC	TACAGGCTTGTCACTCGAATT
**ACAN**	CTGAACGACAGGACCATCGAA	CGTGCCAGATCATCACCACA
**COL2A1**	CACTCAAGTCCCTCAACAACCAG	GGGGTCAATCCAGTAGTCTCCAC

Movie S1. Motion of mPDA-C@M under PBS (Brownian motion). (MP4)

Movie S2. Motion of mPDA-C@M under H_2O_2 solution. (MP4)

Movie S3. Motion of mPDA-C@M under PBS with NIR irradiation. (MP4)

Movie S4. Motion of mPDA-C@M under H_2O_2 solution with NIR irradiation. (MP4)

Movie S5. Ultrasound detection of mPDA-C@M-treated OA joint. (MP4)
